# Musculoskeletal ultrasound for treating rheumatoid arthritis to target—a systematic literature review

**DOI:** 10.1093/rheumatology/keac261

**Published:** 2022-05-04

**Authors:** Ettore Silvagni, Sara Zandonella Callegher, Eleonora Mauric, Sofia Chiricolo, Nikolaus Schreiber, Annarita Tullio, Alen Zabotti, Carlo Alberto Scirè, Christian Dejaco, Garifallia Sakellariou

**Affiliations:** Rheumatology Unit, Department of Medical Sciences, Università degli Studi di Ferrara and Azienda Ospedaliero-Universitaria S. Anna, Cona, FE; Department of Rheumatology, Hospital of Brunico (SABES-ASDAA), Brunico, BZ; Division of Rheumatology, IRCCS Policlinico San Matteo Foundation, University of Pavia, Pavia, PV, Italy; Division of Rheumatology, IRCCS Policlinico San Matteo Foundation, University of Pavia, Pavia, PV, Italy; Department of Rheumatology, Medical University of Graz, Graz, Austria; Institute of Epidemiology; Rheumatology Clinic, Department of Medical Area, Academic Hospital ‘Santa Maria della Misericordia’, Udine, UD; Rheumatology Unit, School of Medicine and Surgery, University of Milano-Bicocca; Epidemiology Unit, Italian Society for Rheumatology, Milan; Department of Rheumatology, Hospital of Brunico (SABES-ASDAA), Brunico, BZ; Department of Rheumatology, Medical University of Graz, Graz, Austria; Istituti Clinici Scientifici Maugeri, University of Pavia, Pavia, PV, Italy

**Keywords:** RA, imaging, ultrasound, diagnosis, prognosis, prediction, systematic literature review

## Abstract

**Objective:**

We aimed to systematically review the literature to retrieve evidence on the diagnostic and prognostic value of musculoskeletal ultrasound for a treat to target (T2T) approach in RA.

**Methods:**

Eight research questions were developed addressing the role of ultrasound (including different ultrasound scores and elementary lesions) for diagnosis, monitoring and prognosis of RA. PubMed and EMBASE were searched (2005–2020). Articles on RA and reporting data on musculoskeletal ultrasound were included and extracted according to the underlying questions, and risk of bias assessed according to the study design.

**Results:**

Out of 4632 records, 60 articles were included. Due to clinical heterogeneity, meta-analysis was not possible. Ultrasound better predicted disease relapses with respect to clinical examination in patients in remission, while both methods performed similarly in predicting response to therapy, achievement of remission and radiographic progression. Ultrasound was superior to clinical examination in diagnosing joint involvement using another imaging modality, such as magnetic resonance imaging, as reference. Limited ultrasound scores performed like more extensive evaluations for the detection of joint inflammation and for outcome prediction. Higher ultrasound scores of synovitis were linked to poor outcomes at all disease stages, but a specific cut-off distinguishing between low- and high-risk groups did not emerge.

**Conclusions:**

These data confirm the pivotal role of ultrasound when evaluating synovial inflammation and when identifying RA patients at higher risk of relapse. Further research is needed to better define the role of ultrasound in a T2T management strategy in moderately-to-highly active RA.

Rheumatology key messages

- Musculoskeletal ultrasound confirmed its value in diagnosing synovitis and predicting relapse.
- Reduced ultrasound scores perform comparably to the extensive ones.
- Knowledge on the impact of ultrasound applied to RA management is still scarce.

## Introduction

In recent years, the management of RA has undergone major progress, given the availability of new drugs, early diagnosis, prompt treatment initiation, and treat-to-target (T2T) strategy [1–4]. Specifically, treating disease to target is recommended by EULAR and other international guidelines, since its adoption leads to improved clinical, functional and imaging outcomes [2, 5]. One crucial aspect of T2T is to regularly measure disease activity using clinical composite scores. These scores, however, may lead to over- or underestimation of joint inflammation, given that non-inflammatory pain (e.g. painful comorbidities) may result in high scores whereas subclinical synovitis is not sufficiently captured [6–9].

Musculoskeletal ultrasound is more sensitive and specific than clinical examination in detecting synovial inflammation; nonetheless, its role in the follow-up of RA patients is controversial [10]. Ultrasound is increasingly used in clinical practice for diagnosis and follow-up of these patients [11]; however, clinical trials adopting a T2T strategy by incorporating imaging as a parameter in early RA patients failed their primary endpoints [12–14]. The main objective of these trials was to test whether a strategy aiming at clinical plus imaging remission was superior to a conventional strategy targeting clinical remission alone. While there was a trend towards better radiographic outcomes in the clinical plus imaging groups, the primary clinical endpoints, e.g. achievement of clinical remission, were not met. The value of imaging as part of T2T in patients with elevated clinical scores, in which imaging might help to distinguish between true joint inflammation and non-inflammatory causes of increased scores, has not been investigated yet.

The present systematic literature review (SLR) was conducted in preparation of a clinical trial promoted by the Italian Society of Rheumatology (SIR). The aim of this trial will be to investigate the value of ultrasound as part of a T2T strategy in RA patients with doubtful clinical inflammation as compared with clinical evaluation alone, trying to exploit the possibility of correctly identifying inflammatory *vs* non-inflammatory conditions by ultrasound. Herein, we summarize the evidence regarding the value of ultrasound compared with other imaging to detect active inflammation and ultrasound *vs* clinical assessment to predict different outcomes in RA. We also investigate the performance of different ultrasound composite scores, ultrasound elementary lesions and grades of lesions for monitoring and outcome prediction of RA patients.

## Methods

The SLR was conducted following the PRISMA 2020 Checklist (Supplementary Table S1, available at *Rheumatology* online) [15]. The areas of interest covered the value of ultrasound, including different scores and lesions, in the diagnosis of active inflammation compared with different imaging modalities, different ultrasound scores and different ultrasound lesions, as well as in the prediction of clinical, radiographic and functional outcomes compared with clinical assessment. Eight research questions were developed and transformed into PICOs (Patients, Intervention, Comparator, Outcome, Study type) (Table 1; Supplementary Table S2, available at *Rheumatology* online), sharing pre-defined inclusion and exclusion criteria.

**Table 1:** Research areas and key clinical questions driving the search strategies and the inclusion criteria

Research area	Key clinical questions
Prognostic and diagnostic value of ultrasound	In patients with RA, what is the value of ultrasound *vs* clinical examination to predict outcome?
	In patients with RA, what is the diagnostic value of ultrasound for active inflammation as compared with clinical examination, using other imaging as reference standard?
Value of ultrasonographic scores	In patients with RA, what is the diagnostic value of different ultrasound scores (A *vs* B) to detect inflammation using extensive ultrasound assessment as reference standard?^a^
	In patients with RA, what is the diagnostic value of different ultrasound scores (A *vs* B) to detect inflammation using clinical examination as reference standard?^a^
	In patients with RA, what is the value of different ultrasound scores (A *vs* B) to predict outcome?^a^
Value of ultrasound-detected elementary lesions	In patients with RA, what is the value of ultrasound lesion A *vs* ultrasound lesion B to predict outcome?
	What is the diagnostic value of ultrasound lesion A *vs* lesion B for the diagnosis of active RA as compared with OA?^a^
Value of the grading of ultrasound-detected elementary lesions	In patients with RA, what is the value of level of ultrasound lesion A *vs* B for outcome?^a^

a The results for this research question have been reported in the Supplementary Material, available at *Rheumatology* online.

Search strategies were applied to PubMed and EMBASE (1 January 2005–21 June 2020) (Supplementary Table S3, available at *Rheumatology* online). Searches were performed by one author (G.S.) and limited to humans, adults and English language. The timespan was chosen to assess comparable equipment. Records were imported into bibliographic management software (Zotero) and duplicates removed. Five investigators (S.C., E.M., E.S., N.S., S.Z.C.) performed screening, selection, data extraction and bias assessment, working in five pairs comprising different combinations of reviewers to assess titles and abstracts. Pairs of reviewers assessed the eligibility of the full-texts. Only studies including at least 10 patients with RA were included. RA populations of interest included: early RA, longstanding RA, RA patients on conventional synthetic/biologic/targeted synthetic DMARDs, RA patients in clinical remission or with active disease. All study designs (including letters reporting original data) were eligible except case reports, editorials and narrative reviews. Disagreement between reviewers was resolved by consensus within the pairs. Another investigator (G.S.) was involved as a tiebreaker if consensus could not be obtained. Data from included articles were extracted in pre-specified forms, including general information on the article, features of the population and, when available, 2 × 2 tables of diagnostic accuracy, odds ratios (OR) and risk ratios with 95% CI. For diagnostic studies, the outcomes of interest were the diagnosis of inflammatory lesions and of active disease, while for predictive studies, the outcomes were clinical response, clinical relapse, joint damage on imaging, disability and maintenance of treatment. Risk of bias (RoB) of included studies was assessed with the Newcastle–Ottawa scale for cohort and case–control studies [16], the Cochrane RoB (RoB2) for randomized-controlled trials (RCTs), and the Quality Assessment of Diagnostic Accuracy Studies (QUADAS-2) tool for diagnostic studies [17]. Results are presented in evidence tables. Meta-analysis was not performed due to clinical heterogeneity.

## Results

**Fig. 1:**
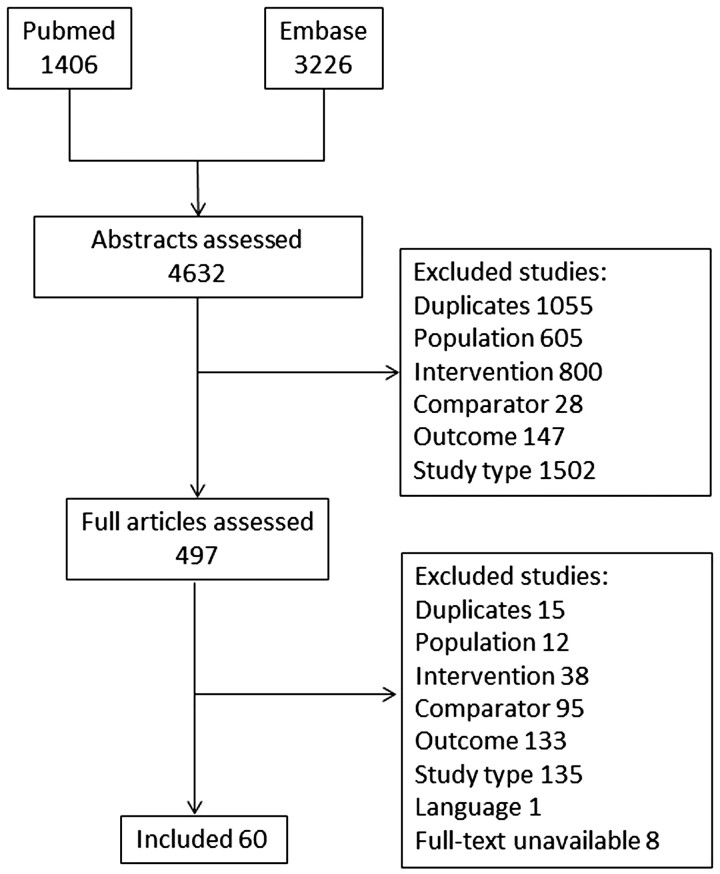
Flowchart showing the selection process

**Fig. 2:**
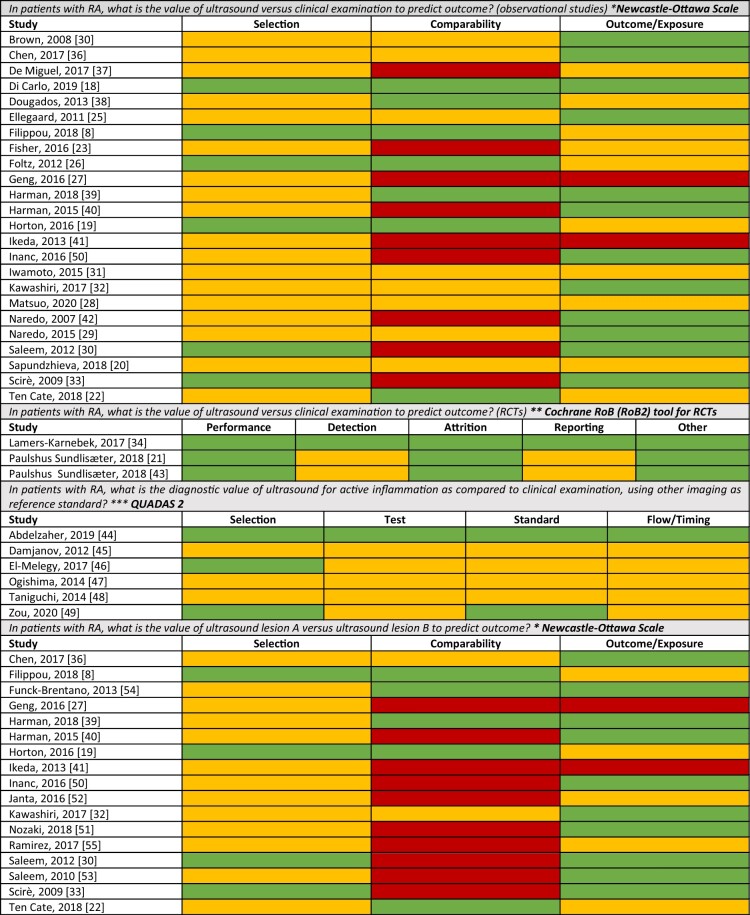
Risk of bias assessment The risk of bias was defined through different tools, depending on study design. For the Newcastle–Ottawa scale: for selection red corresponds to ≤1 star, yellow to 2–3 stars, green to 4 stars; for comparability, red corresponds to 0 stars, yellow to 1, green to 2; for exposure/outcome red corresponds to ≤1 star, yellow to 2 stars, green to 3 stars. For the QUADAS 2: for selection, red corresponds to 3 no, yellow to 1 yes or at least 1 unclear, green to 3 yes; for test: red corresponds to 2 no, yellow to 1 yes or at least 1 unclear, green to 2 yes; for standard, red corresponds to 2 no, yellow to 1 yes or at least 1 unclear, green to 2 yes; for flow/timing: red corresponds to 3 no, yellow to 1–2 yes or at least 1 unclear, green to 3 yes. For RCTs: red corresponds to low risk of bias, yellow to some concerns on the risk of bias, red to high risk of bias. *Risk of bias assessed through the Newcastle-Ottawa scale; **Risk of bias assessed through the Cochrane tool for RCTs; ***Risk of bias assessed through the QUADAS. RCT: randomized controlled trial. Colour version available online.

Of 4632 articles evaluated, 60 studies were finally included (Fig. 1; Supplementary Figs S1 and S2, available at *Rheumatology* online). There were four RCTs, 40 prospective cohorts, 15 cross-sectional studies and one retrospective observational study. Studies referring to research questions about composite scores and grades of ultrasound lesions, as well as the diagnostic role of ultrasound for RA *vs* OA are summarized in the Supplementary Material, available at *Rheumatology* online. The risk of bias assessment is presented in Fig. 2 and in Supplementary Table S4, available at *Rheumatology* online.

### Value of ultrasound compared with clinical examination to predict future outcomes

**Fig. 3:**
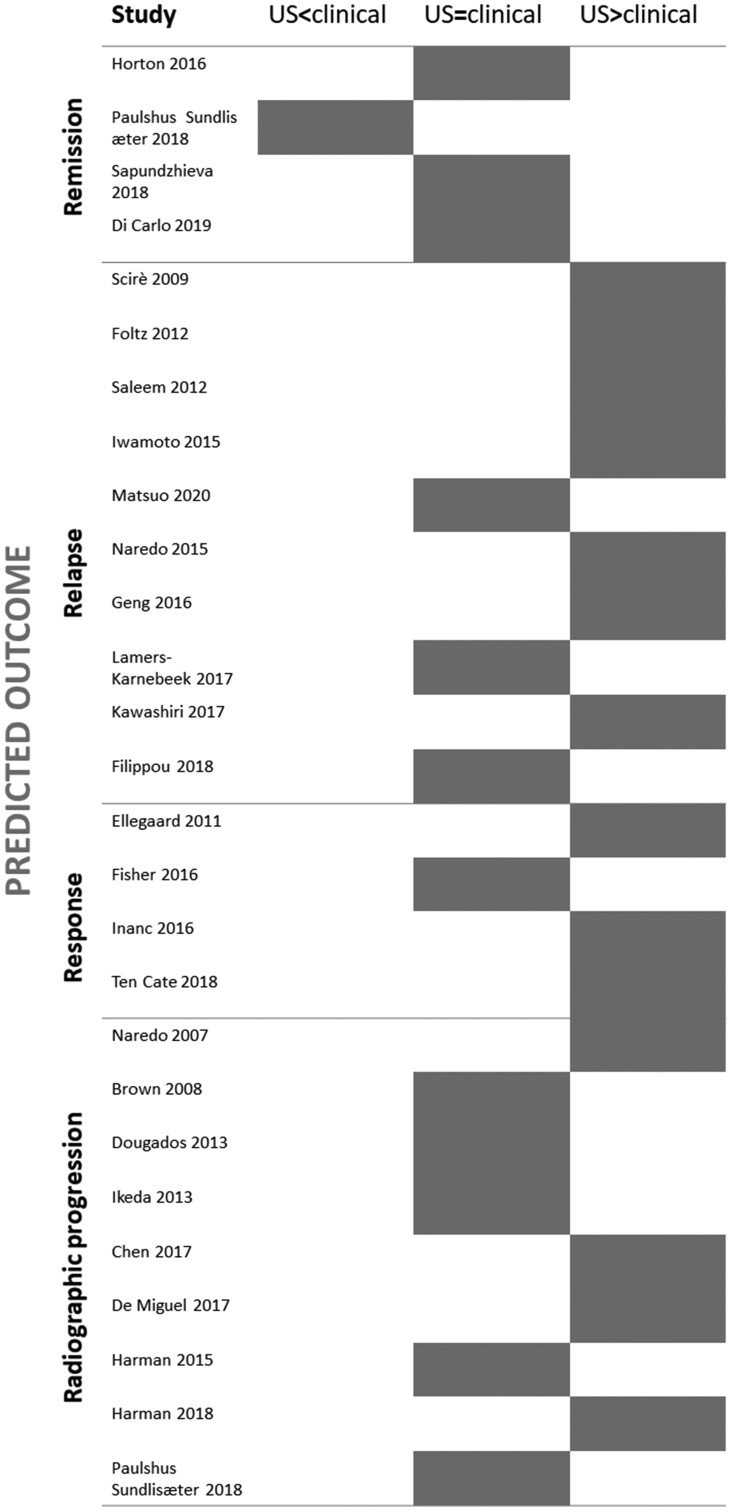
Summary of studies evaluating ultrasound *vs* clinical examination to predict outcomes The main finding of the studies (ultrasound being inferior, equal or superior to clinical examination for predicting outcomes) is represented by the grey box. Studies on remission and response were performed in patients with active disease, while those on relapse in patients in clinical remission. Radiographic progression was assessed both in studies on clinical remission and active disease. US: ultrasound.

Studies are summarized in Fig. 3 and Supplementary Table S5, available at *Rheumatology* online. The risk of bias of included studies is summarized in Fig. 2.

#### Clinical remission

Three prospective studies [18–20], and one RCT [21], including 598 patients, compared ultrasound with clinical examination for the prediction of clinical remission. In the RCT (ARCTIC), DMARD-naïve early RA patients were followed up for 2 years by tight control aiming for clinical remission (DAS in 44 joints with ESR <1.6). In half of patients, clinical plus ultrasound remission [no joints with power Doppler (PD) signal] was targeted. Failure to achieve sustained DAS remission at 16–24 months was predicted in both univariate and multivariate analysis only by a higher tender joint count (TJC) [OR (95% CI) 0.90 (0.86, 0.94), *P* < 0.001] but not by ultrasound parameters [21]. Horton *et al.* reported that in early RA neither ultrasound nor DAS28-CRP predicted remission [19], while in the other two studies in patients with long-lasting RA both clinical examination and ultrasound predicted remission to a similar extent [18, 20]. Of note, the TaSER study was not included in the present SLR, as it did not fulfil the inclusion criteria. In fact, it enrolled a mixed population of RA and undifferentiated arthritis, and separate results for RA patients were not available [14].

In summary, in the ARTIC, only TJC predicted remission at 16–24 months, while in prospective studies there were no differences between clinical examination and ultrasound in this regard.

#### Response to therapy

Four prospective studies including 345 patients explored the predictive value of ultrasound *vs* clinical parameters for treatment response [22–25], while in two studies, ultrasound was not of additional value over clinical examination alone [22, 23]. Ellegaard *et al.* found that PD, quantified by the colour fraction in the wrist, was the only predictor of maintenance of an anti-TNFα after 1 year in patients with long-lasting RA and moderate–high disease activity [25]. Conversely, Inanc *et al.* reported that a higher baseline 28-joints PD score was the only parameter which, in multivariate analysis, predicted a lack of EULAR response to TNFα blockers at 3 months [OR (95% CI) 0.86 (0.75–0.98)] in patients with long-lasting, biologic-naïve RA and high baseline disease activity [24].

Globally, ultrasound and clinical examination had a similar value in predicting response to therapy in active RA.

#### Clinical relapse

Ten studies, including 1240 subjects, compared the values of ultrasound and clinical examination for predicting clinical relapse of patients in remission at 6 or 12 months. Eight of these concluded that ultrasound outperformed clinical examination in both longstanding [26–32] and early RA [33]. In five articles, PD positivity was the best predictor of relapse in multivariate analysis among several clinical and ultrasound parameters, yielding ORs between 3.08 and 13.9 [26–29, 33]. In the study by Iwamoto *et al.*, both PD and grey scale (GS) synovitis (GSS) outperformed clinical parameters [31], while according to Saleem *et al.* both PD and disability (Health Assessment Questionnaire) were independently associated with increased risk of flare [31]. In the study by Kawashiri *et al.* the presence of ultrasound-detected bone erosions predicted relapse within 12 months after treatment discontinuation [OR (95% CI) 8.35 (1.78, 53.2)] [32]. In contrast to these results, in a secondary analysis of the STARTER study (the only analysis of the study eligible for inclusion in this research question), the addition of ultrasound to clinical variables did not improve the prediction of future flares in patients with baseline DAS28 < 3.2 [8]. An RCT (POET-US) in longstanding RA with low disease activity revealed that ultrasound had limited value to predict a flare after the discontinuation of anti-TNFα when added to clinical parameters [34].

In summary, 8 out of 10 studies demonstrated that PD-positive synovitis outperformed clinical examination in predicting flares at 6 or 12 months in longstanding and early RA patients in clinical remission.

#### Radiographic progression

Eight prospective studies [35–42] and one RCT [43], enrolling 880 patients, compared ultrasound and clinical examination for predicting radiographic progression. In two studies, a low PD score in early RA predicted the absence of radiographic progression at 12 months better than the swollen joint count (SJC) and the TJC [39, 42]. In the study by de Miguel *et al.* the absence of PD but not clinical indexes predicted the lack of radiographic progression at 12 months in longstanding RA [37]. Chen *et al.* reported that delayed improvement of GSS one month after starting an anti-TNFα more accurately predicted 1-year radiological damage than clinical measures such as DAS28 [36]. In four studies, X-ray progression at 6, 12 and 24 months was similarly predicted by ultrasound and clinical parameters [35, 38, 40, 41]. In the sub-analysis of the ARCTIC RCT, ACR/EULAR Boolean remission, absence of PD signal and minimal GSS (sum score ≤2 of 0–96 joints) performed similarly for the prediction of no radiographic progression from 12–24 months after baseline [ACR/EULAR Boolean remission: OR (95% CI) 3.2 (1.2, 8.4); absence of PD signal: OR (95% CI) 3.6 (1.3, 10.0); minimal GSS: OR (95% CI) 3.2 (1.2, 8.0)] in a cohort of moderately active early RA patients [43]. However, in the main analysis of the ARTIC study, there was a borderline statistically significant difference at 24 months concerning the change of radiographic joint damage, favouring the ultrasound-guided group.

In summary, absence of ultrasound-detected inflammation seems to have a slight superiority over absence of clinically detected inflammation regarding radiographic progression at 12–24 months.

### Value of ultrasound compared with clinical examination to detect active inflammation, using other imaging as reference standard

Six studies, including 286 patients, addressed this issue [44–49] (Table 2; Supplementary Table S6, available at *Rheumatology* online), four with a cross-sectional [44, 46, 48, 49] and two with a prospective design. The risk of bias of the included studies is reported in Fig. 2 [45, 47]. In all the studies, ultrasound was superior to clinical examination in detecting inflammatory lesions, using MRI as the reference. In three studies, ultrasound performed better than clinical examination of swollen joints at wrists and MCP joints to identify active inflammation in longstanding RA, in terms of area under the receiver operating characteristic curve (AUC) (AUC for sonographic identification of synovitis/effusion 0.65–0.75 *vs* clinical examination 0.36–0.55), sensitivity (ultrasound 0.27–0.64 *vs* clinical examination 0.31–0.52), specificity (ultrasound 0.95–1 *vs* clinical examination 0–0.98), positive predictive value (ultrasound 0.65–1 *vs* clinical examination 0.45–0.86) and negative predictive value (ultrasound 0.41–0.88 *vs* clinical examination 0.78–0.86) [45, 47, 48]. In the remaining three studies, ultrasound was superior to clinical examination in detecting inflammatory lesions in shoulders, temporomandibular and MTP joints [44, 46, 49].

**Table 2:** Studies on the diagnostic value for active inflammation compared with clinical examination

Study	Population	Study design	Site	Reference standard	Results	
Abdelzaher, 2019 [44]	RA with shoulder pain	Cross-sectional	Shoulders	MRI	LHB tenosynovitis	
					Se 0.87	
					Sp 0.98	
					SAD bursitis	
					Se 0.72	
					Sp 0.95	
					Subscapularis tenosynovitis	
					Se 0.92	
					Sp 0.98	
Damjanov, 2012 [45]	Longstanding RA with active disease	Prospective cohort	MCPs, wrists	MRI	US synovitis/effusion AUC (95% CI)	Clinical synovitis/effusion AUC (95% CI)
					Wrists 0.75 (0.43, 1)	Wrist 0.36 (0.04, 0.68)
					MCP 0.65 (0.53, 0.76)	MCP 0.55 (0.43, 0.66)
El-Melegy, 2017 [46]	Longstanding RA	Cross-sectional	TMJ joint	MRI	US abnormalities were less frequent than MRI abnormalities (77.5% *vs* 82.5%), in particular regarding erosions. US assessment was positive in 77.5% of cases, while clinical examination in 67.5%	
Ogishima, 2014 [47]	Longstanding RA	Prospective cohort	MCP, PIP, wrists	MRI	Ultrasound	Clinical examination
					Se (95% CI) 0.48 (0.42, 0.54)	Se (95% CI) 0.43 (0.37, 0.49)
					Sp (95% CI) 0.95 (0.92, 0.95)	Sp (95% CI) 0.87 (0.84, 0.89)
Taniguchi, 2014 [48]	Longstanding RA	Cross-sectional	MCPs, wrists	MRI	Ultrasound	Clinical examination
					Wrist	Wrist
					Se (95% CI) 0.64 (0.49, 0.77)	Se (95% CI) 0.52 (0.37, 0.66)
					Sp (95% CI) 1 (0.73, 1)	Sp (95% CI) 0 (0, 0.26)
					MCP	MCP
					Se (95% CI) 0.27 (0.18, 0.38)	Se (95% CI) 0.31 (0.21, 0.41)
					Sp (95% CI) 1 (0.98, 1)	Sp (95% CI) 0.98 (0.95, 0.99)
Zou, 2020 [49]	Early RA	Cross-sectional	MTPs 2–5	MRI	Ultrasound	Clinical examination
					GS	Se (95% CI) 0.28 (0.18, 0.39)
					Se (95% CI) 0.59 (0.47, 0.70)	Sp (95% CI) 0.90 (0.82, 0.95)
					Sp (95% CI) 0.35 (0.24, 0.46)	
					PD	
					Se (95% CI) 0.69 (0.48, 0.85)	
					Sp (95% CI) 0.58(0.49, 0.67)	

AUC: area under the receiver operating characteristic curve; GS: grey scale; PD: power Doppler; Se: sensitivity; Sp: specificity; TMJ: temporo-mandibular joint.

In summary, ultrasound was superior to clinical examination in detecting inflammatory lesions in different joints, with MRI used as reference.

### Value of different elementary ultrasound lesions to predict future outcomes

**Fig. 4:**
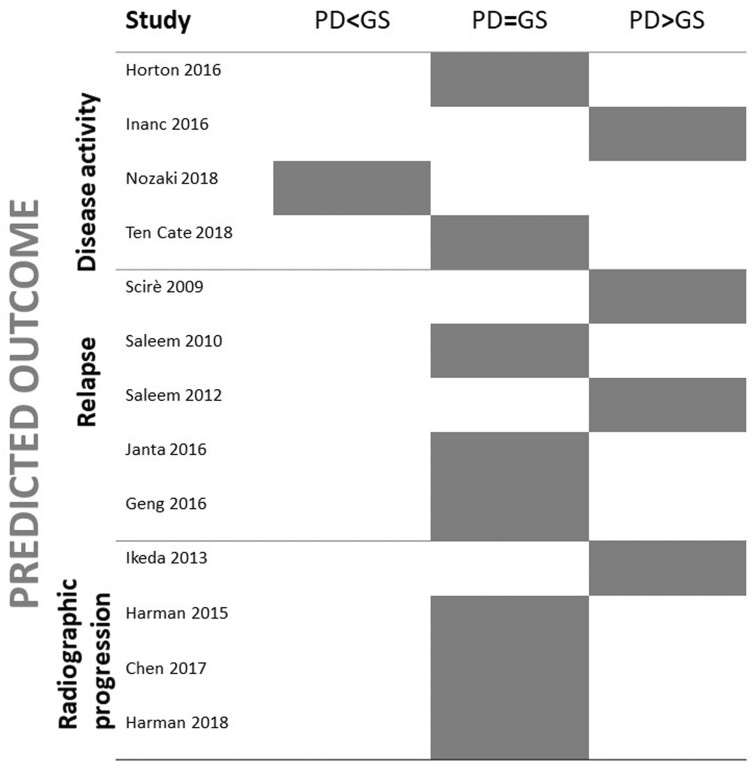
Summary of studies evaluating the value of lesion A *vs* lesion B to predict outcome The main finding of the studies (power Doppler being inferior, equal or superior to grey scale for predicting outcomes) is represented by the grey box. Studies on disease activity included patients with active disease, while those on relapse were performed in clinical remission. Radiographic progression was assessed both in studies on clinical remission and in studies on active disease. GS: grey scale; PD: power Doppler.

Seventeen studies evaluated the role of specific ultrasound lesions for outcome prediction (Fig. 4; Supplementary Table S11, available at *Rheumatology* online). Global risk of bias of the included studies is reported in Fig. 2.

#### Clinical response

Among the five studies, including 475 patients, assessing clinical response, two [19, 22] did not find any association between GSS and PD synovitis and different definitions of clinical remission at 12 months in early RA. In the work by Harman *et al.* [40] instead, time-integrated values for GSS, PD-positive synovitis, GS tenosynovitis and PD-positive tenosynovitis were linked to DAS44 at 1 year. Among patients starting an anti-TNFα, Inanc *et al.* [24] highlighted that clinical responders at 3 months had higher PD score at baseline; this was not observed for GSS. Nozaki *et al.* did not confirm these results in a 54-week study, suggesting that a synovial hypertrophy index, defined as the sum of the synovial hypertrophy scores for each joint, predicted clinical response, while PD did not [50].

Overall, it appears that GSS and PD synovitis might be more useful in predicting clinical remission in long-standing RA, while their role in early RA is still questionable.

#### Clinical relapse

Studies were selected when a comparison among different elementary lesions was available, independently from clinical evaluation; therefore, the main conclusions differ slightly from the previous research question. Seven studies enrolling 736 patients assessing clinical relapse were included. Data concerning the association of GS and PD scores with the risk of flare are heterogeneous, with some studies reporting that simultaneous PD-positive tenosynovitis and PD-synovitis predicted flare in long-standing RA [adjusted OR (95% CI) 2.09 (1.06, 4.13)] [8], while others reported that PD-positive synovitis better predicted flare than GSS [30, 33, 51], even though statistical significance was not always reached [27, 52]. Other researchers reported no value of GSS or PD synovitis for predicting flares [32]. One possible source of bias is that, in some studies, it is unclear if ultrasound influenced treatment decisions or not [30, 52].

Globally, the risk of relapse in RA patients in remission was higher for PD-positive synovitis than for GS-synovitis, despite some exceptions.

#### Radiographic progression

Seven studies, including 726 patients, evaluated radiographic progression at 6–18 months. In one prospective study of patients starting anti-TNFα [36], baseline GSS and PD did not predict radiographic progression at 1 year, while the modification of GSS score at 1 month did, with patients yielding no improvement in GSS experiencing higher structural damage than those improving. Similarly, the cumulative accrual of PD scores at 12 and 24 weeks compared with baseline was associated with radiographic progression at 24 weeks in RA patients starting methotrexate [41]. In another study on early RA [40], GSS and PD synovitis at baseline, but not GS and PD tenosynovitis, predicted radiographic progression at 1 year [53]. Similarly, baseline GSS, PD-synovitis and ultrasound-detected erosions predicted radiographic progression at 1 year [39], but none of these lesions performed better than another. In two studies on patients in remission, radiographic progression did not correlate with baseline GS and PD scores, while structural deterioration occurred more frequently in patients who fulfilled a more stringent definition of ultrasound synovitis (including simultaneous synovial hyperplasia ≥2 and PD or concurrent presence of GS tenosynovitis and synovitis) than in those not fulfilling it [8, 54]. In summary, data on the comparison of different ultrasound lesions to predict radiographic progression in RA is heterogeneous. While in early RA, baseline GSS and PD-synovitis predicted radiographic progression, this could not be confirmed for long-standing RA.

The results of the research questions ‘Value of restricted ultrasound scores to detect inflammation, using extensive ultrasound assessment as reference standard’, ‘Value of different ultrasound scores to detect inflammation, using clinical examination as reference standard’, ‘Value of different ultrasound scores to predict future outcomes’**,‘**Value of different elementary ultrasound lesions for diagnosis of active RA *vs* osteoarthritis’ and ‘Value of grade of different elementary ultrasound lesions to predict future outcomes’ are depicted in the Supplementary File and Supplementary Tables S7–S14, available at *Rheumatology* online.

## Discussion

The exact role of musculoskeletal ultrasound in guiding treatment decisions in RA has not been fully clarified yet [11, 55, 56]. The objectives of this SLR were to evaluate studies comparing ultrasound and clinical examination to predict outcomes of interest for RA patients, as well as its value to detect active inflammation. Globally, ultrasound, in particular PD, is helpful to identify active disease, and enables the prediction of clinical outcomes, mainly in patients in clinical remission. Data remain heterogeneous in the context of moderate-to-high disease activity, and hence further research is necessary in this patients’ group.

Sixty studies were retrieved, mostly observational, pertinent to all research questions. Since the main aim was to assess the value of ultrasound *vs* clinical examination to predict specific outcomes, we analysed its role in general and, more specifically, the role of specific scores, elementary lesions and grades. Relating to the first research question of our SLR (i.e. the value of ultrasound *vs* clinical examination to predict outcomes of interest), PD-positive synovitis might be more helpful than clinical examination in predicting flares in clinical remission. In fact, 8 out of 10 studies demonstrated that ultrasound performed better than clinical scores, especially PD-positive synovitis, suggesting that sonography can help clinicians to decide about how to manage patients in remission. EULAR recommendations for the use of imaging in RA [10], developed almost 10 years ago, suggested a role of ultrasound in monitoring disease activity, without clarifying how much, to which joints, and to which population sonography should be applied. Our study helps to select the population (i.e. RA patients in clinical remission) in which ultrasound is of highest value. ARCTIC and TaSER trials, which included ultrasound in the treatment strategy [12–14], failed their primary endpoints, and DMARDs should probably not be increased or started *de novo* in early RA patients who have already reached clinical remission. Concerning the most relevant question, whether treatment should be de-escalated once clinical remission has been achieved, ultrasound can provide important insights. Patients without clinical activity but PD synovitis, for example, are at a higher risk of flare and radiographic progression, and it would probably not be wise to step down treatment in this group; rather, these patients should undergo close clinical (and maybe also imaging-based) monitoring.

Globally, ultrasound and clinical evaluation had a similar value in predicting clinical response in active RA, and no information can be inferred regarding a possible role for sonography in personalized treatment. Since the only RCT [21] was performed in patients with early DMARDs-naïve RA, no RCT specifically addressed our main research question on whether RA patients with active disease despite effective treatment could benefit of a US-guided approach to clarify the main treatment strategy to select. We also want to highlight the limitations of the ARCTIC and TaSER trials (the latter was not included in this SLR because it did not meet the inclusion criteria), like the absence of blinding of the ultrasound findings chosen for decisions-making or the included population. These limitations prevent the generalization to all RA patients of the conclusions of these studies [57]. Moreover, an RCT on ultrasound in a T2T strategy should bridge the discrepancy between clinical perception of the disease status and index-based evaluation of disease activity. We also lack specific information on distinct disease-populations (e.g. oligo-articular or mono-articular involvement in RA) in which ultrasonography might be applied for a T2T strategy. With respect to structural outcomes, our SLR retrieved some evidence suggesting a slight superiority of ultrasound over clinical evaluation for the prediction of radiographic progression. In particular, absence of ultrasound inflammation was associated with lower radiographic damage. Our results confirm the suggestion to use ultrasound by EULAR [10], but should be replicated in RCTs.

In line with EULAR recommendations [10, 58, 59], we confirmed that ultrasound was superior to clinical examination in detecting active inflammation using MRI as reference. Ultrasound revealed a higher sensitivity detecting subclinical inflammation in a considerable proportion of patients, and also a higher specificity by excluding inflammation in patients with tenderness but no swelling. This provides the basis to study the value of ultrasound in moderately active RA in order to inform treatment decisions and potentially to prevent overtreatment of patients.

Another question refers to the minimum joint count that should be scanned by sonography. In our SLR (see Supplementary Results, available at *Rheumatology* online) ultrasound scores ranged from 3 to 44 joints, but none of this was validated for clinical practice. Most studies were performed in longstanding RA, leaving areas of uncertainty in early phases of the disease and in remission. Globally, simplified scores gave similar information to extensive ones in terms of synovitis detection, while erosive damage was tendentially underestimated by the former. Restricted scores outperformed clinical examination in the detection of synovial inflammation to a similar extent as extensive scores, indicating that these limited ultrasound scores could be a valid, less time consuming alternative for clinical practice.

We then assessed the role of specific lesions in predicting clinical outcomes. The risk of flare in remission tended to be higher in patients with PD-positive synovitis than in those with GS-synovitis only. Since some sonographic tools like the GLOESS [60] contemplate high cumulative scores even in the absence of PD, the separate evaluation of PD may be considered when such scores are used for studies and/or clinical practice. The role of specific ultrasound lesions in predicting response and/or radiographic progression in active RA is uncertain and should be evaluated further. In early RA patients, baseline GS, PD and erosions predicted subsequent radiographic progression, but none of these lesions attained superiority over the others [39, 53].

With respect to the diagnostic question concerning elementary lesions, we found that high-grade GSS, PD-positive synovitis, erosions and tenosynovitis were all helpful in discriminating RA from other diseases, particularly OA, acknowledging that the number and quality of studies were low. Finally, the majority of studies assessing the grade of ultrasound lesions highlighted that higher grade of PD-synovitis performed better than lower scores in predicting outcomes of interest (clinical response, radiographic progression) [10].

Our study has some limitations, which partly relate to those of the primary studies. First, most research was performed using ultrasound in ‘non-realistic’ settings, mainly by interpreting sonographic findings independently from clinical evaluation. While this might be required for blinding, in clinical routine, ultrasound is mostly used as an extension of clinical examination, particularly in doubtful situations. The risk of bias of the included studies was generally moderate, in particular concerning exposure and comparability, and this affected the overall quality of the SLR itself. Third, there was a broad clinical heterogeneity among the studies. This could be explained by the attempt of investigators to clarify a specific research question rather than replicating another study.

In conclusion, ultrasound assessment, and in particular PD-positive synovitis, outperformed clinical examination concerning the prediction of relapse and radiographic progression in the setting of clinical remission. Moreover, since sonography outperformed clinical examination in detecting active disease, more research is needed to clarify the value of ultrasound in predicting clinical response to therapy or selection of adequate treatment, as well as to elucidate the role of sonography in a T2T strategy. This could be of value specifically in cases of moderately active disease, a clinical context in which composite indexes confirm several gaps [1, 9]. Here, sonography might serve as a tiebreaker in avoiding overtreatment.

## Supplementary Material

keac261_Supplementary_Data

## Data Availability

The authors have full control of all primary data and agree to allow the journal to review data if requested.
