# Supplementary material for: Musculoskeletal ultrasound for treating rheumatoid arthritis to target—a systematic literature review
Source: Rheumatology (Oxford). 2022 May 4;61(12):4590–602. doi: 10.1093/rheumatology/keac261 (PMC9707059; doi:10.1093/rheumatology/keac261)
Supplement: keac261_Supplementary_Data [file keac261_supplementary_data.zip › keac261_Supplementary_Data/rhe-21-3180-File006.docx]

**Musculoskeletal ultrasound for treating rheumatoid arthritis to target – a systematic literature review. Supplementary Online Material.**

Supplementary Table S2: Research questions and corresponding PICOs, driving the literature search and the inclusion/exclusion of the articles.

| **Research Area** | **Research Question** | **Population** | **Intervention** | **Comparator** | **Outcome** | **Study type** |
| --- | --- | --- | --- | --- | --- | --- |
| **Research area A**  ***In which RA population might ultrasound be of greatest value to guide treatment decisions?*** | *In patients with RA, what is the value of ultrasound versus clinical examination to predict outcome?* | Adult patients with RA, including:   - Early RA - Longstanding RA - Patients in csDMARDs - Patients in b/tsDMARDs - Patients in clinical remission - Patients with active disease | Musculoskeletal US (joints, periarticular structures) | Clinical examination of the joints/periarticular structures | - Clinical response using different criteria and composites - Imaging determined joint damage - Clinical relapse using different criteria and composites - Disability - Maintenance of current DMARD therapy | cohort studies  case-control studies  randomized clinical trials  systematic literature reviews  meta-analyses |
|  | *In patients with RA, what is the diagnostic value of ultrasound for active inflammation as compared to clinical examination, using other imaging as reference standard?* | Adult patients with RA, including:   - Early RA - Longstanding RA - Patients in csDMARDs - Patients in b/tsDMARDs - Patients in clinical remission - Patients with active disease | Musculoskeletal US (joints, periarticular structures) AND Clinical examination of the joints/periarticular structures | Other imaging:  MRI  CT  PET scan  Other imaging able to assess directly inflammation | Diagnosis of synovitis, effusion, tenosynovitis | cohort studies  case-control studies  randomized clinical trials  systematic literature reviews  meta-analyses |
| **Research area B**  ***Which ultrasound composite score should be used to guide treatment decisions in RA?***  ***Which scores have been validated so far?***  ***Is it sufficient to investigate only clinically active/suspected joints or is a joint composite preferable?*** | *In patients with RA, what is the diagnostic value of different ultrasound scores (A vs. B) to detect inflammation using extensive ultrasound assessment as reference standard?* | Adult patients with RA, including:   - Early RA - Longstanding RA - Patients in csDMARDs - Patients in b/tsDMARDs - Patients in clinical remission - Patients with active disease | Ultrasound score A | Ultrasound score B (the most extensive ultrasound score applied in the study) | Diagnosis of synovitis, effusion, tenosynovitis | cohort studies  case-control studies  randomized clinical trials  systematic literature reviews  meta-analyses |
|  | *In patients with RA, what is the diagnostic value of different ultrasound scores (A vs. B) to detect inflammation using clinical examination as reference standard?* | Adult patients with RA, including:   - Early RA - Longstanding RA - Patients in csDMARDs - Patients in b/tsDMARDs - Patients in clinical remission - Patients with active disease | Ultrasound score A AND ultrasound score B | Clinical examination of joints and tendons | Diagnosis of synovitis, effusion, tenosynovitis | cohort studies  case-control studies  randomized clinical trials  systematic literature reviews  meta-analyses. |
|  | *In patients with RA, what is the value of different ultrasound scores (A vs. B) to predict outcome?* | Adult patients with RA, including:   - Early RA - Longstanding RA - Patients in csDMARDs - Patients in b/tsDMARDs - Patients in clinical remission - Patients with active disease | Ultrasound score A | Ultrasound score B | - Clinical response using different criteria and composites - Imaging determined joint damage - Clinical relapse using different criteria and composites - Disability - Maintenance of current DMARD therapy | cohort studies  case-control studies  randomized clinical trials  systematic literature reviews  meta-analyses |
| **Research area C**  ***Which elementary lesions might be useful to predict outcomes in RA?*** | *In patients with RA, what is the value of ultrasound lesion A versus ultrasound lesion B to predict outcome?* | Adult patients with RA, including:   - Early RA - Longstanding RA - Patients in csDMARDs - Patients in b/tsDMARDs - Patients in clinical remission - Patients with active disease | Ultrasound lesion A | Ultrasound lesion B | - Clinical response using different criteria and composites - Imaging determined joint damage - Clinical relapse using different criteria and composites - Disability - Maintenance of current DMARD therapy | cohort studies  case-control studies  randomized clinical trials  systematic literature reviews  meta-analyses |
|  | *What is the diagnostic value of ultrasound lesion A versus lesion B for the diagnosis of active RA as compared to OA?* | Adult patients with RA, including:   - Early RA - Longstanding RA - Patients in csDMARDs - Patients in b/tsDMARDs - Patients in clinical remission - Patients with active disease | Ultrasound lesion A AND ultrasound lesion B | Clinical diagnosis of active diseases | Diagnosis of active RA vs diagnosis of OA | cohort studies  case-control studies  randomized clinical trials  systematic literature reviews  meta-analyses |
| **Research area D**  ***Which grade of elementary lesions might be useful to predict outcomes in RA?*** | *In patients with RA, what is the value of level of ultrasound lesion A versus B for outcome?* | Adult patients with RA, including:   - Early RA - Longstanding RA - Patients in csDMARDs - Patients in b/tsDMARDs - Patients in clinical remission - Patients with active disease | Ultrasound lesion of a definite grade | Ultrasound lesion of a different grade compared to the tested grade | - Clinical response using different criteria and composites - Imaging determined joint damage - Clinical relapse using different criteria and composites - Disability - Maintenance of current DMARD therapy | cohort studies  case-control studies  randomized clinical trials  systematic literature reviews  meta-analyses |

Supplementary Table S3: Search strategies applied in PubMed and Embase.

| **Key words used for the systematic search in Pubmed**  **#1** Arthritis, Rheumatoid [Mesh]  **#2** arthritis, rheumatoid [All fields]  **#3** rheumatoid arthritis [All fields]  **#4** OR 1-3  **#5** Ultrasonography [Mesh]  **#6** Ultrason*[All fields]  **#7** Ultrasound[All fields]  **#8** Sonograph*[all fields]  **#9** Ecograph*[all fields]  **#10** Echotomograph*[all fields]  **#11** ultrasonograph*[all fields]  **#12** OR 5-11  **#13** 1 AND 12  Filters: Publication date from 2005/01/01; Humans; English; Adult: 19+ years |
| --- |
| **Key words used for the systematic search in Embase**  **#1** 'rheumatoid arthritis'/exp  **#2** 'ultrasound'  **#3** ultrasonograph*  **#4** 'echography'/exp  **#5** 'echography'  **#6** OR 2-5  **#7** 1 AND 6  Filters: AND ([adult]/lim OR [middle aged]/lim OR [aged]/lim OR [very elderly]/lim) AND [humans]/lim AND [english]/lim AND [embase]/lim AND [2005-2020]/py |

**Supplementary Results**

*Value of different ultrasound scores to detect inflammation, using extensive ultrasound assessment as reference standard*

Twelve studies involving 1235 patients, whose features are summarized in Table S7, evaluated the value of different restricted ultrasound scores to detect inflammation using extensive assessment of joints as reference standard. Most studies were performed in patients with longstanding RA starting cs-DMARDs or b-DMARDs, while 2 of them were on patients in clinical remission. RoB was globally moderate (Table S4). Specifically, while the information inferred by included articles relating to flow and timing of assessments permitted to exclude a possible source of bias, the description of patients’ selection, tests and standards were burdened by higher risks of bias. Different scores were evaluated, with the number of assessed joints ranging from 3 to 44, and with a significant heterogeneity across studies. The most frequently assessed joints in simplified scores were the wrists and the 2^nd^ MCP joints, while shoulders, ankles and elbows were less commonly included.

The sensitivity of simplified scores was moderate to high for synovitis detection, while erosive damage was tendentially under-recognized. Specifically, a 12-joints model including elbows, wrists, 2^nd^ and 3^rd^ MCPs, knees, and ankles had a sensitivity of 100% and 94% in detecting patients with at least 1 joint yielding GSS or PD synovitis, respectively, as compared to a 44 joints count [1]. A score combining bilateral wrist, 2^nd^-5^th^ MCP, ankle, and MTPs, as well as a 12-joints assessment, showed the highest correlations with the comprehensive 44-joints ultrasound score among patients in clinical remission [2]. Also, the evaluation of both wrists and 2^nd^ MCP of the dominant hand had a sensitivity of 90% as compared to a 18-joints score among 40 RA patients in DAS28 remission, of whom half had ultrasound-verified subclinical synovitis [3]. Other studies analysed the concordance between different scores in terms of construct validity (evaluated using the Cronbach α coefficient) [4], Spearman’s correlation [5–8], or others [9–11] reporting significant correlations. In summary, both simplified and more extensive scores were equally able to intercept relevant synovial hypertrophy (SH) or PD-positive synovitis; conversely, simplified scores (range 4 to 18 joints examined) underestimated erosive damage in up-to 30% of cases, when compared to more extensive scores (22 joints) [12].

*Value of different ultrasound scores to detect inflammation, using clinical examination as reference standard*

Three studies including 148 patients assessed the value of specific ultrasound scores to detect inflammation using clinical examination as reference standard [4,5,7] (Table S8). Ultrasound scores, comprehensive as well as simplified ones, were moderately able to capture clinical perception of disease activity.

*Value of different ultrasound scores to predict future outcomes*

The role of different ultrasound composite scores in predicting outcomes was investigated in 3 studies (Table S9, Table S10). Specifically, prediction of clinical response, relapses, and radiographic progression were assessed in one study each. Ellegaard et al. [13] evaluated clinical response at one year in longstanding RA patients starting anti-TNFα. A semiquantitative scoring system for PD synovitis at bilateral wrist joints performed poorly in measuring decrease in DAS28, similarly to the quantitative assessment of colour fraction of PD signal [13]. Regarding radiographic progression, Fukae et al. 2010 [14] demonstrated a good correlation between different scores for PD synovitis detection (semiquantitative, quantitative) at baseline and radiographic progression at 20 weeks in early RA patients starting csDMARDs. Longitudinal improvement in quantitative PD at MCPs negatively correlated with radiographic progression at MCPs, but this was not the case at PIP joints using semiquantitative PD assessment. Finally, Janta et al. [15] demonstrated that PD-positive synovitis (across different scores ranging from 6 to 44 joints assessed) was able to identify instable remission (increase in DAS28 or substantial changes in RA treatment) among longstanding RA patients in remission at study entry.

*Value of different elementary ultrasound lesions for diagnosis of active RA versus osteoarthritis*

Five studies including 424 RA patients (Table S12) evaluated the value of ultrasound lesions for differential diagnosis of RA as compared to osteoarthritis (OA) and other non-inflammatory conditions[9,16–19]. Despite the low number of studies included, the risk of bias was moderate for selection of studies and standard description, while it was low for test and timing assessment (Table S4). GSS grade 3, PD-positive synovitis, erosions, and tenosynovitis were all helpful to discriminate between RA and non-inflammatory diseases, but the heterogeneity of included studies precludes formal comparisons. The type of joint affected by ultrasound synovitis (e.g. wrist, MCP in the case of inflammatory conditions, distal interphalangeal joints in the case of OA) helped to distinguish between the two conditions, as well. [17,19].

*Value of grade of different elementary ultrasound lesions to predict future outcomes*

We identified 5 studies assessing 112 patients on this topic, one RCT [20] and 4 prospective studies [21–24] (Table S13, Table S14). Most studies assessed hands and wrists only [21–23], the risk of bias was moderate-high.

Two studies reported that the higher the grade of PD at baseline, the higher the risk of clinical joint swelling at the subsequent visit [20,22]. In the RCT, which involved early RA patients with active disease randomized to a T2T strategy with or without ultrasound, the OR of subsequent joint swelling increased progressively for each grade of PD (range 0-3) as compared to no PD activity (p<0.001) [20]. Conversely, no effect of different ultrasound sum scores and cut-offs on the prediction of persistence of active disease at 1 year was found by Ten Cate *et al.* [24].

Raffeiner *et al.* reported that in RA patients in remission, a PD grade 3 was associated with a RR of 3.49 (p<0.05) for radiographic progression and a PD grade 2 with a RR of 4.58 (p<0.01), while PD grade 1 showed no association (RR 1.58, p=0.16) [23]. Fukae *et al*. reported that joints with positive synovial vascularity (i.e. quantitative assessment of PD by counting the number of vascular flow pixels in the region of interest) in longstanding RA patients with active disease were at higher risk of radiographic progression (p≤0.01) irrespective of the extent of vascularity [21].

Supplementary Figure S1. Included studies subdivided for each research question.


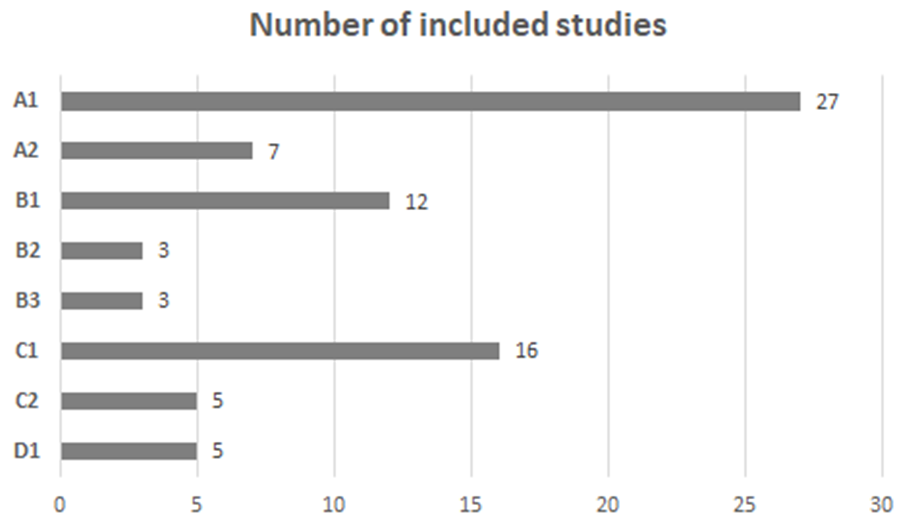


List of research questions: **A1:** in patients with RA, what is the value of ultrasound versus clinical examination to predict outcome?; **A2:** in patients with RA, what is the diagnostic value of ultrasound for active inflammation as compared to clinical examination, using other imaging as reference standard?; **B1:** in patients with RA, what is the diagnostic value of different ultrasound scores (A vs. B) to detect inflammation using extensive ultrasound assessment as reference standard?; **B2:** in patients with RA, what is the diagnostic value of different ultrasound scores (A vs. B) to detect inflammation using clinical examination as reference standard?; **B3:** in patients with RA, what is the value of different ultrasound scores (A vs. B) to predict outcome?; **C1:** in patients with RA, what is the value of ultrasound lesion A versus ultrasound lesion B to predict outcome?; **C2:** what is the diagnostic value of ultrasound lesion A versus lesion B for the diagnosis of active RA as compared to OA?; **D1:** in patients with RA, what is the value of level of ultrasound lesion A versus B for outcome?

Supplementary Figure S2. Total number of patients from the studies included in each research question


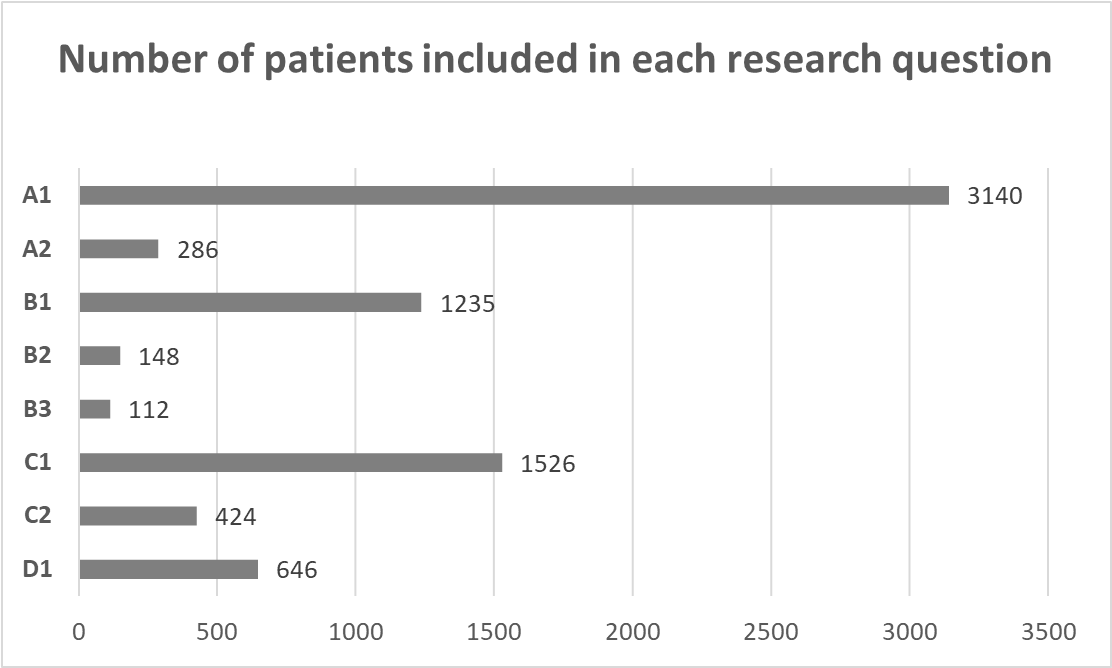


List of research questions: **A1:** in patients with RA, what is the value of ultrasound versus clinical examination to predict outcome?; **A2:** in patients with RA, what is the diagnostic value of ultrasound for active inflammation as compared to clinical examination, using other imaging as reference standard?; **B1:** in patients with RA, what is the diagnostic value of different ultrasound scores (A vs. B) to detect inflammation using extensive ultrasound assessment as reference standard?; **B2:** in patients with RA, what is the diagnostic value of different ultrasound scores (A vs. B) to detect inflammation using clinical examination as reference standard?; **B3:** in patients with RA, what is the value of different ultrasound scores (A vs. B) to predict outcome?; **C1:** in patients with RA, what is the value of ultrasound lesion A versus ultrasound lesion B to predict outcome?; **C2:** what is the diagnostic value of ultrasound lesion A versus lesion B for the diagnosis of active RA as compared to OA?; **D1:** in patients with RA, what is the value of level of ultrasound lesion A versus B for outcome?

Supplementary Table S4. Risk of Bias assessment. The risk of bias was defined through different tools, depending on study design. For the Newcastle-Ottawa scale: for selection red corresponds to ≤1 star, yellow to 2-3 stars, green to 4 stars; for comparability, red corresponds to 0 stars, yellow to 1, green to 2; for exposure/outcome red corresponds to ≤1 star, yellow to 2 stars, green to 3 stars. For the QUADAS 2: for selection, red corresponds to 3 no, yellow to 1 yes or at least 1 unclear, green to 3 yes; for test: red corresponds to 2 no, yellow to 1 yes or at least 1 unclear, green to 2 yes; for standard, red corresponds to 2 no, yellow to 1 yes or at least 1 unclear, green to 2 yes; for flow/timing: red corresponds to 3 no, yellow to 1-2 yes or at least 1 unclear, green to 3 yes. For RCTs: red corresponds to low risk of bias, yellow to some concerns on the risk of bias, red to high risk of bias.

| *In patients with RA, what is the diagnostic value of different ultrasound scores (A vs. B) to detect inflammation using extensive ultrasound assessment as reference standard? **** ***QUADAS 2*** | | | | | | | | | | |
| --- | --- | --- | --- | --- | --- | --- | --- | --- | --- | --- |
| **Study** | **Selection** | | **Test** | | | **Standard** | | | **Flow/Timing** | |
| Dougados, 2009 [4] |  | |  | | |  | | |  | |
| El-Gohary, 2019 [5] |  | |  | | |  | | |  | |
| Fukae, 2009 [9] |  | |  | | |  | | |  | |
| Hammer, 2011 [6] |  | |  | | |  | | |  | |
| Kawahara, 2019 [11] |  | |  | | |  | | |  | |
| Kawashiri, 2011 [7] |  | |  | | |  | | |  | |
| Naredo, 2005 [8] |  | |  | | |  | | |  | |
| Naredo, 2008 [1] |  | |  | | |  | | |  | |
| Naredo, 2013 [2] |  | |  | | |  | | |  | |
| Picchianti Diamanti, 2018 [3] |  | |  | | |  | | |  | |
| Sivakumaran, 2019 [12] |  | |  | | |  | | |  | |
| Tan, 2018 [10] |  | |  | | |  | | |  | |
| *In patients with RA, what is the diagnostic value of different ultrasound scores (A vs. B) to detect inflammation using clinical examination as reference standard? **** ***QUADAS 2*** | | | | | | | | | | |
| **Study** | **Selection** | | **Test** | | | **Standard** | | | **Flow/Timing** | |
| Dougados, 2009 [4] |  | |  | | |  | | |  | |
| El-Gohary, 2019 [5] |  | |  | | |  | | |  | |
| Kawashiri, 2011 [7] |  | |  | | |  | | |  | |
| *What is the diagnostic value of ultrasound lesion A versus lesion B for the diagnosis of active RA as compared to OA? **** ***QUADAS 2*** | | | | | | | | | | |
| **Study** | **Selection** | | **Test** | | | **Standard** | | | **Flow/Timing** | |
| Ehrenstein, 2018 [16] |  | |  | | |  | | |  | |
| Fukae, 2009 [9] |  | |  | | |  | | |  | |
| Glimm, 2016 [17] |  | |  | | |  | | |  | |
| Gok, 2013 [18] |  | |  | | |  | | |  | |
| Hussain, 2018 [19] |  | |  | | |  | | |  | |
| *In patients with RA, what is the value of level of ultrasound lesion A versus B for outcome? (observational studies) ** ***Newcastle-Ottawa Scale*** | | | | | | | | | | |
| Fukae, 2013 [21] |  | | |  | | | |  | | |
| Gartner, 2013 [22] |  | | |  | | | |  | | |
| Raffeiner, 2017 [23] |  | | |  | | | |  | | |
| Ten Cate, 2018 [24] |  | | |  | | | |  | | |
| *In patients with RA, what is the value of level of ultrasound lesion A versus B for outcome? (RCTs)* ***** Cochrane RoB (RoB2) tool for RCTs*** | | | | | | | | | | |
| **Study** | **Performance bias** | **Detection bias** | | | **Attrition bias** | | **Reporting bias** | | | **Other bias** |
| Nordberg, 2018 [20] |  |  | | |  | |  | | |  |

**Summary of findings tables**

Research area A

Supplementary Table S5: In patients with RA, what is the value of ultrasound versus clinical examination to predict outcome?

| **Study** | **N** | **Population** | **Study design** | **Intervention (US technique)** | **Comparator** | **Results** |
| --- | --- | --- | --- | --- | --- | --- |
| Brown, 2008 [25] | 102 | Longstanding RA in remission  Mean symptom duration 7 years | Prospective cohort study | ATL HDI 300 10-15 MHz  A single experienced sonographer blinded to all other study findings  Bilateral MCPs 2-5 and wrist  GS and PD graded 0-3 | DAS28 | ***Radiographic progression – 1 yr***  DAS28  OR (95% CI) 1.54 (0.89, 2.65)  Total US PD score  OR (95% CI) 1.36 (1.02, 1.81)  Dominant Hand US PD score OR (95% CI) 1.64 (1.03, 2.61) |
| Chen, 2017 [26] | 32 | Longstanding RA in TNFis | Prospective cohort study | ESAOTE MyLab70, 6-18 MHz transducer  At each centre, a sonographer performed ultrasound examination, an experienced observer blinded to all other study findings scored the images in random order.  Intraobserver reliability: weighted κ values were median 0.8 for GS synovitis and 0.6 for PDUS.  12 joints (bilateral elbows, wrists, MCP 2-3, PIP 2-3)  GS and PD graded 0-3 | DAS28 | ***Radiographic progression – 1 yr***  Multiple linear regression  DAS28 coefficient 0.13, SE 0.35, p 0.719  No improvement in GS 0-1 month coefficient 0.50, SE 0.22, p 0.036  No improvement in GS 0-3 months coefficient 0.04, SE 0.70, p 0.487  No improvement in PD 0-1 month coefficient 0.04, SE 0.36, p 0.653  No improvement in PD 0-3 months coefficient 0.04, SE 0.29, SE 0.36, p 0.250 |
| De Miguel, 2017 [27] | 357 | RA starting TNFis  Mean (sd) disease duration 7.5 (7.1) years | Prospective cohort study  Missing data: imputation of data not performed | GE Logiq5 GE, Logiq E and Logic9 ESAOTE MyLab25 and MyLab70  At each centre a sonographer blind to clinical data performed ultrasound examination  Acceptable inter-reader reliability for gray scale (Kendall W = 0.653; P < 0.0005) and PD (Kendall W = 0.762; P < 0.0005)  12-joint US score (bilateral elbow, wrist, second and third MTPs, knee, and ankle)  GS and PD | DAS28CRP | ***Radiographic progression – 1 yr***  PD- vs PD+  Baseline: OR (95% CI) 0.197 (0.046,0.861)  6 months: OR (95% CI) 0.134 (0.047,0.378)  PD<1.5 vs PD≥1.5  Baseline: OR (95% CI) 0.313 (0.117, 0.835)  6 months: OR (95% CI) 0.312 (0.142,0.710)  No significant association between baseline DAS28CRP and 1-year progression. |
| Di Carlo, 2019 [28] | 130 | RA starting ABT  Mean (sd) disease duration 11.21 (8.76) years | Prospective cohort study | Equipment and sonographer not specified  Score: US synovitis weighted according to the Thompson's articular index, then normalized to a 0–10 scale  Joints: wrists, second and third MCP joints, second and third PIP joints  GS and PD | Self-TJC | ***Response***  Logistic regression using the “responders” category (modified Residual Minimal Disease Activity and/or Boolean criteria) as dependent variable:  US-score (0–10) coefficient: −0.103631; SE: 0.047453, p=0.0211  Self-TJC (0–10) coefficient: −0.183666; SE: 0.096377, p=0.0412 |
| Dougados, 2013 [29] | 59 | RA starting TNFis  Mean (sd) disease duration 10±8 years | Prospective cohort study | ESAOTE Technos MPX, ESAOTE MyLab, TOSHIBA APLIO, PHILIPS HD11, BK Mini Focus  At each centre an experienced sonographer blind to clinical data performed ultrasound examination  Joints: MCP, PIP, wrists and MTP joints  GS and PD 0-3 | Clinical assessment of synovitis | ***Radiographic progression – 2 yr***  In joints with no clinical synovitis:  GS OR (95%CI) 2.16 (1.16,4.02)  PD OR (95%CI) 3.50 (1.77,6.95)  In joints with normal US: clinical detection of synovitis OR 2.79 (1.35,5.76) |
| Ellegaard, 2011 [30] | 109 | RA on TNFis  Mean (sd) disease duration 10.4 (9.0) years | Prospective cohort study | Siemens  Four experienced sonographers, two investigators with long-standing experience in image evaluation performed all image analyses blinded to the patients’ clinical characteristics  Image acquisition technique has shown a test–retest reliability intraclass correlation coefficient (ICC) of 0.77  Wrist joint  PD, scored by the square-root of colour fraction of PD | TJC and SJC | ***Response – 1 yr***  (Defined as maintenance of treatment)  Square root of CF predicted treatment maintenance 0.484 (0.188) in completers of follow-up vs 0.363 (0.215) in non-persisting patients, p= 0.008.  CF predicted treatment maintenance (0.270 (0.180) in completers of follow-up vs 0.176 (0.151) in non-persisting patients, p= 0.013).  TJC did not predict treatment maintenance 11 (8) in completers of follow-up vs 10 (10) in non-persisting patients, p= 0.321.  SJC did not predict treatment maintenance 8 (6) in completers of follow-up vs 8 (8) in non-persisting patients, p= 0.486. |
| Filippou, 2018 [31] | 340 | RA in clinical remission  Mean (sd) disease duration 9.75 (8.07) years | Prospective cohort study  Missing data were handled using available case analysis; in each analysis, all cases with available data on the relevant variables were included | ESAOTE MyLab 70XVG, ESAOTE MyLab Twice, GE Logiq9, GE LogiqE9  Ultrasonographers were rheumatologists expert in musculoskeletal ultrasound.  Good to excellent reliability (weighted kappa ≥0.7) was required before selection for the study.  22 joints (wrists, MCPs, PIPs), 22 tendons  GS and PD at joints (0-3) and tendons (0-3) | TJC and SJC | ***Disease flare – 1yr***  PD+ synovitis and tenosynovitis:  AUC (95%CI) 0.690 (0.626, 0.754)  Concurrent GS+ synovitis and tenosynovitis:  AUC (95%CI) 0.680 (0.618, 0.742)  SJC  AUC (95%CI) 0.665 (0.602, 0.728)  TJC  AUC (95% CI) 0.661 (0.598, 0.725) |
| Fisher, 2016 [32] | 23 | Longstanding RA starting TNFis  Mean (sd) disease duration 10 (4.15) years | Prospective cohort study | Esaote MPX Technos  Sonographers’experience and reliability not specified  MCPs  GS (synovial thickness) and PD 0-3 | DAS28 | ***Response -1 yr***  DAS28 and ultrasonographic measurements at week 4 were used to predict good response at 1 year: no predictor was statistically significant. |
| Foltz, 2012 [33] | 85 | RA in remission/LDA | Prospective cohort study | ESAOTE Technos  Two experienced sonographers blind to clinical data  Good interreader reliability  14 joints  Lesions: GS and PD, erosions 0-3 | DAS28 | ***Relapse – 1 yr***  Baseline PD-positive synovitis count:  adj OR (95% CI) 6.3 (2.0,20.3)  Baseline DAS did not predict flare. |
| Geng, 2016 [34] | 126 | RA in clinical remission  Mean (sd) disease duration 5.2 (6.1) years | Prospective cohort study | ESAOTE Mylab 90  Two experienced rheumatologists blinded to all clinical findings performed US.  22 joints (Bilateral wrists, MCP1-5, PIP 1-5)  GS, PD, tenosynovitis, erosions 0-3 | DAS28 | ***Relapse – 1 yr***  PD>0 at baseline:  OR (95% CI) 8.8 (2.7, 28.4)  DAS28 at baseline:  OR (95% CI) 2.8 (1.0, 2.0) |
| Harman, 2018 [35] | 48 | Early RA  Mean (sd) symptom duration 3.12 (1.33) months | Prospective observational study | GE LOGIQ P5  A trained ultrasonographer with 4 years of experience performed US.  GS, PD, tenosynovitis 0-3 | DAS28 | ***Radiographic progression - 18 months***  Multivariate analysis  Baseline total GSUS synovitis score: B:0.091 beta:0.417 p=0.011  1-month total GSUS synovitis score: B:0.518 beta:0.549 p=0.028  3-months total GSUS synovitis scores: B:2.261 beta:1.110 p=0.278  Baseline total PDUS synovitis scores: B:0.057 beta:0.476 p=0.015  1-month total PDUS synovitis scores: B:0.075 beta:0.358 p=0.017  3-months total PDUS synovitis score: B:1.154 beta:0.214 p=0.507  Baseline DAS28: B:4.646 beta:4.324 p=0.293  1-month DAS28: B:-1.190 beta:-0.096 p=0.453  3-month DAS28: B:-4.852 beta:-0.366 p=0.453 |
| Harman, 2015 [36] | 68 | Early RA  Mean (sd) symptom duration 0.63 (0.61) years | Prospective cohort study  Available data from four patients with incomplete follow-up were excluded from the study. | GE LOGIQ P5  Two trained ultrasonographers with 1 and 3 years of experience performed US.  Interobserver agreement was 87, 91, 77, and 92 % for the presence/absence of GSUS synovitis, PDUS synovitis, GSUS tenosynovitis, and PDUS tenosynovitis with κ values of 0.70, 0.72, 0.64, and 0.78, respectively.  GS, PD, tenosynovitis, erosions 0-3 | TJC, SJC, DAS44 | ***Response – 1 year***  (Defined as DAS at 1 year)  Correlation between time-integrated value of clinical and US parameters and disease DAS44  Baseline TJC: 0.35 (p<0.05)  Baseline SJC: 0.31 (p<0.05)  Baseline DAS44: 0.79 (p<0.001)  Baseline total GSUS synovitis scores: 0.38 (p<0.01)  Baseline total PDUS synovitis scores: 0.39 (p<0.01)  Baseline total tenosynovitis scores: 0.36 (p<0.01)  Baseline total PD tenosynovitis scores: 0.41 (p<0.01)  ***Radiographic progession – 1 yr***  (Defined as total radiographic score progression)  Correlation between time-integrated value of clinical and total radiographic score progression  Baseline TJC: 0.33 (p<0.05)  Baseline SJC: 0.35 (p<0.05)  Baseline DAS44: 0.37 (p<0.05)  Baseline total GSUS synovitis scores: 0.54 (p<0.01)  Baseline Total PDUS synovitis scores: 0.56 (p<0.001)  Baseline total tenosynovitis scores: not significant  Baseline total PD tenosynovitis scores: not significant |
| Horton, 2016 [37] | 105 | Early RA on cs-DMARDs  Median (IQR) symptom duration 6 (4-13) months | Prospective cohort study  To determine any difference between patients included in analyses and those excluded due to missing data, Chi-squared tests (or Fisher’s exact tests) for categorical variables, t-tests for continuous variables following a normal distribution and Mann-Whitney U tests for non-parametric variables were performed | GE Logiq E9  A trained sonographer blinded to the clinical findings  26 joints  GS and PD 0-3 | SJC, DAS28CRP | ***Remission – 1 yr***  (Defined based on DAS28CRP, DAS44CRP, Boolean definition)  *Prediction of DAS28CRP<2.6*  Baseline SJC28 OR (95% CI) 0.98 (0.89, 1.08) NS  Baseline DAS28-CRP OR (95% CI) 0.66 (0.45, 0.97) p=0.04  Total GS score OR (95% CI) 1.00 (0.96, 1.04) NS  Total PD score OR (95% CI) 0.98 (0.92, 1.05) NS  *Prediction of DAS44CRP<1.6*  Baseline SJC28 OR (95% CI) 0.97 (0.87, 1.07) NS  Baseline DAS28CRP OR (95% CI) 0.57 (0.38, 0.86) p=0.008  Total GS score OR (95% CI) 0.98 (0.94, 1.02) NS  Total PD score US OR (95% CI) 0.97 (0.91, 1.04) NS  *Prediction of Boolean remission*  Baseline SJC28 OR (95% CI) 1.00 (0.87, 1.14) NS  Baseline DAS28CRP OR (95% CI) 0.53 (0.30, 0.95) p=0.03  Total GS score OR (95% CI) 1.00 (0.95, 1.05) NS  Total PD score OR (95% CI) 0.98 (0.89, 1.07) NS |
| Ikeda, 2013 [38] | 69 | RA starting MTX or bDMARDs  Median (IQR) disease duration 35 (17–111) months | Prospective cohort study | GE LOGIQ 7 Pro, GE LOGIQ E9, Toshiba Viamo, Hitachi HI VISION Avius  Two rheumatologists trained for musculoskeletal ultrasound, who were blinded to clinical and laboratory data  ICC for intraobserver reliability of GS and PD scores were very high (0.93 and 0.97, respectively). ICC for interobserver reliability of GS and PD scores were high (0.85 and 0.89, respectively)  GS and PD 0-3 | DAS28CRP | ***Radiographic progression – 24 weeks***  Correlation (Spearman’s correlation coefficient) between cumulative scores (sum of net weights at baseline, 12 weeks, 24 weeks) radiographic progression at 24 weeks  Cumulative DAS28-CRP: 0.342 (p=0.009)  Cumulative total GS score: 0.062 (p=0.649)  Cumulative total PD score: 0.357 (p=0.006) |
| Inanc, 2016 [39] | 39 | Longstanding RA starting 1^st^-line TNFis  Mean disease duration 9.14 years | Prospective cohort study | ESAOTE MyLab70  Sonographers’experience and reliability not specified28 joints  GS and PD 0-3 | DAS28 | ***Response – 3 months***  (Defined as EULAR response)  Baseline DAS28 in responders (mean (SD)): 5.6 (1.0)  Baseline DAS28 in non-responders (mean (SD)): 5.6 (1.4); p=0.94  Baseline PD score in responders (mean (SD)): 13.0 (5.1)  Baseline PD score in non-responders (mean (SD)): 20.6 (11.8); p=0.035  Baseline GS score in responders (mean (SD)): 16.4 (6.9)  Baseline GS score in non-responders (mean (SD)): 24.9 (14.4); p=0.054 |
| Iwamoto, 2015 [40] | 42 | Longstanding RA in clinical remission stopping biologics  Mean (sd) disease duration 82 (6.7) | Prospective cohort study | Toshiba Aplio XG, Viamo, Hitachi HI VISION Avius, or HI VISION Ascendus  Six rheumatologists trained for musculoskeletal ultrasound rheumatologists trained for musculoskeletal ultrasound  ICC for intraobserver reliability of GS and PD scores were very high (0.94 and 0.99, respectively). ICC for interobserver reliability of GS and PD scores were high (0.86 and 0.89, respectively)  DAS28 joints, ankles, MTPs  GS and PD 0-3 | DAS28 | ***Relapse – 6 months***  DAS28≥1.5  AUC (95%CI) 0.55 (0.37,0.73)  Se 0.81  Sp 0.38  GS score≥14  AUC (95%CI) 0.76 (0.60,0.91)  Se 0.50  Sp 0.92  PD score≥3  AUC (95%CI) 0.73 (0.56,0.91)  Se 0.50  Sp0.96 |
| Kawashiri, 2017 [41] | 40 | Longstanding RA in clinical remission or LDA, stopping biologics  Mean (sd) disease duration 3.5 (5.5) | Prospective cohort study | Toshiba Aplio 500, Hitachi Ascendus, Hitachi Noblus, GELOGIQ E9, multifrequency linear transducer (12–14 MHz)  Five rheumatologists trained for musculoskeletal ultrasound (Japan College of Rheumatology-certified sonographers) performed US.  22 joints (bilateral wrist 1-5 MCP, 1-5 PIP  GS and PD 0-3  Positivity ofGS and PD in articular synovitis was defined as a score ≥1 for GS and PD.  Bone erosions 0-1 | SDAI at initiation of bDMARDs | ***Relapse – 1 yr***  SDAI at initiation of bDMARDs  OR (95%CI)  0.95 (0.88,1.03)  Bone erosions  OR (95%CI)  8.35 (1.78,53.2) |
| Lamers-Karnebek, 2017 [48] | 259 | Longstanding RA in clinical remission or LDA  Median disease duration 9 years | Randomized clinical trial  The model was developed on complete cases instead of using imputation on the full dataset | 22 joints (MCP 1-5, wrists, MTP 2-5)  18 experienced ultrasonographers (16 rheumatologists, 1 radiologist and 1 rheumatologist in training) blinded for clinical data  The reliability was optimized by training and calibration sessions  GS and PD 0-3 | Model without US findings | ***Disease flare – 1 yr***  (Defined as increase in DAS28≥0.6 with DAS28≥3.2)  At least one joint with US inflammation: HR (95%CI)  1.7 (1.1, 2.5)  The discriminative ability of both models was comparable and modest: c-index(95%CI) 0.62  (0.56,0.68) without US and 0.64 (0.59,0.70) with US. |
| Matsuo, 2020 [42] | 212 | Longstanding RA in clinical remission or LDA | Prospective cohort | Aplio 500 with 12 MHz probe  Sonographers’experience and reliability not specified  The 28 joints (MCP2-5), bilateral wrist, MTP2-5, bilateral Lisfranc, bilateral cuneonavicular, bilateral Chopart, and bilateral ankle  GS 0-3  SMI | DAS28 | **Disease flare – 2 yr**  DAS28-CRP (+ 1) 1.48 0.63–3.46  Wrist SMI score ≧ 1 3.08 1.56–6.08 |
| Naredo, 2007 [43] | 42 | Early RA who started cs-DMARDs therapy  Mean (± SD) disease duration 6.8 (± 3.6) months (range 1.5–12) | Prospective cohort study | GE Logiq 500CL  multifrequency linear array transducers (7–12 MHz)  Single rheumatologist experienced in ultrasound who was unaware of the clinical, laboratory, and radiographic findings and who was not involved in the treatment decisions  Intraobserver kappa values for the US evaluation of each joint ranged from good to excellent (k= 0.75–1), intraobserve overall agreement ranged from 98% to 100%. Intraobserver ICC was 0.99 (95% CI 0.99 – 0.99) for the ultrasound joint count for active synovitis and 0.99 (95% CI 0.98 – 0.99) for the ultrasound joint index for power Doppler signal  28 joints (bilateral glenohumeral, elbow, wrist, MCP, PIP of the hands, and knee)  Presence of joint synovitis (intraarticular effusion and/or synovial hypertrophy) and intraarticular PD graded 0-3  Active synovitis defined as the presence of intraarticular synovitis with PD. | TJC and SJC | ***Radiographic progression – 1 yr***  Time-integrated values vs 1-year increase in total SHS. Pearson’s or Spearman’s correlation  TJC 0.36  SJC 0.46  DAS28 0.40  USJCAS 0.61  USJPD 0.59  ***DAS28 – 1 yr***  Time-integrated values vs 1-year DAS28. Pearson’s or Spearman’s correlation  TJC 0.50  SJC 0.45  DAS28 0.75  USJCAS 0.63  USJPD 0.63  ***Disability (HAQ) – 1 yr***  Time-integrated values vs 1-year HAQ. Pearson’s or Spearman’s correlation  TJC 0.50  SJC ns  DAS28 0.49  USJCAS ns  USJPD ns |
| Naredo, 2015 [44] | 77 | Long-standing RA patients in sustained clinical remission. Biological therapy was tapered according to an agreed strategy  Mean (S.D., range) disease duration: 13.1 (6.8, 3-31) years | Prospective observational cohort study | Mylab 70 XVG; Esaote, Genoa, Italy with 6- to 18-MHz transducer for superficial areas and a 4- to 13-MHz transducer for deep areas.  Single rheumatologist experienced in ultrasound who was unaware of the clinical, laboratory, and radiographic findings and who was not involved in the treatment decisions  42 joints  Presence of joint synovitis and intraarticular PD graded 0-3. A global index for Doppler synovitis (DSI) (the sum of synovial power Doppler signal scores obtained for each evaluated joint; 0-108) | DAS28 | ***Biologic therapy tapering failure at 6 months*** *(multivariate logistic models)*  DSI > 0: OR 13.91 (3.44- 56.29)  DAS28 >2.2: OR 11.27 (2.79-45.54)  ***Biologic therapy tapering failure at 12 months*** *(multivariate logistic models)*  DSI > 0: OR 29.92 (6.81- 131.40)  DAS28 >2.2: OR 5.81 (1.62-20.93) |
| Paulshus Sundlisæter, 2018 ARD [49] | 103 | DMARDs naive early RA patients.  Mean disease duration (±SD): 6.7 (±5.3) months | RCT  Missing radiographs were imputed by inter- or extrapolation if a minimum of 2 radiographs were available, whereas missing clinical, laboratory or ultrasound variables at the follow-up visits were imputed by interpolation | Siemens Antares or GE Logiq E9 with linear probes (11.4/13.0 MHz)  Experienced sonographers  32 joints  GS and PD scored 0-3, calculated total GS (0-96) and PD (0-96) scores  US remission defined as: no PD signal and minimal GS synovitis (sum score ≤2) | SJC | ***Radiographic progression – 2 yr***  No radiographic progression (ΔSHS=0) 12-24 months in US remission vs US no remission:  RR (95% CI) 2.31 (1.13,4.70).  RX progression 12-24 months in swollen vs not swollen joints:  RR (95% CI) 1.42 (0.78,2.59). |
| Paulshus Sundlisæter, 2018 Rheumatology [50] | 222 | DMARDs naive early RA patients  Median (range) symptoms duration 5.8 (2.9-10.4) months | RCT  Missing outcome measures were imputed with worst outcome, with robustness analyses in patients with complete datasets | Siemens Antares or GE Logiq E9 with linear probes (11.4/13.0 MHz)  Experienced sonographers  32 joints  GS and PD scored 0-3, calculated total GS (0-96) and PD (0-96) scores | RAI (0-78) and SJC (0-44) | ***Sustained clinical remission – 2 yr***  Sustained ACR/EULAR Boolean remission:  RAI: OR 0.93(0.87,0.98)  SJC: OR 1.00 (0.95,1.04)  GS score: OR 1.00 (0.98,1.02)  PD score 0-96: OR 1.02 (0.99,1.05)  Sustained SDAI remission:  RAI: OR 0.93 (0.89,0.98)  SJC: OR 1.01 (0.97,1.04)  GS score 0-96: OR 1.00 (0.98,1.02)  PD score 0-96: OR 1.01 (0.98,1.04).  Sustained no swollen joint:  RAI: OR 0.95 (0.91,0.98)  SJC: OR 0.96 (0.93,1.00)  GS score: OR 0.98 (0.97,1.00)  PD score: OR 0.99 (0.97,1.02)  Sustained DAS remission at 16-24 months:  RAI: OR 0.90 (0.86,0.94)  SJC: OR 1.01 (0.98,1.05)  GS score: OR 1.01 (0.99,1.03)  PD score: OR 1.00 (0.98,1.03)  Sustained DAS remission, no swollen joint and no radiographic progression:  RAI: OR 0.91 (0.86,0.96)  SJC OR 0.95 (0.91,0.99)  GS score 0-96: OR 0.95 (0.93,0.98)  PD score: OR 0.97 (0.94,1.00) |
| Saleem, 2012 [45] | 93 | Longstanding RA patients in clinical remission (no flares of disease in the last 6 months, stable treatment with cs-DMARDs for 6 months and no indication for a change in treatment)  Mean disease duration (95% CI) 7.0 (4.5–9.5) | Prospective cohort study | Phillips ATL HDI 3000 with a 10–5 MHz ‘hockey stick’ transducer  A single experienced ultrasonographer who was blinded to all other study findings  GS synovial hypertrophy and PD scored according to MCP 2–5 and wrist of the dominant hand, scanned from dorsal and palmar aspects | TJC and SJC | ***Disease flare – 1 yr***  (Increase in disease activity requiring an initiation, change or increase in therapy)  PD score >0  OR (95% CI) 4.08 (1.26,13.19), p=0.014  GS score >0  OR (95% CI) 1.44 (0.28,7.32), p=0.658  TJC OR (95% CI) 1.11 (0.43,2.86), p=0.827  SJC OR (95% CI) 1.21 (0.47,3.10), p=0.690 |
| Sapundzhieva, 2018 [46] | 141c-DMARDs group: 78  b-DMARDs group: 63 | Longstanding RA treated with cs- or b-DMARDs, with moderate or high disease activity according to DAS28-CRP  Median (range) disease duration, months: c-DMARDs group 36 (0–540); b-DMARDs group 96 (0–564) | Prospective cohort study | MyLab 7, Esaote, with a multi-frequency linear probe (10–18 MHz)  A single assessor who was blinded to the patients’ clinical data  US7-score (wrist, 2-3 MCP and PIP, 2-5 MTP  PD synovitis, overall score 0-39 | DAS28 | ***DAS28 clinical remission – 1 yr***  (Spearman’s correlation)  c-DMARDs group: Baseline DAS28: rs = − 0.516, p < 0.001  Baseline PDUS score: rs = − 0.517, p < 0.001  b-DMARDs group: Baseline DAS28: strong negative association rs = − 0.516, p < 0.001  PDUS score rs = − 0.393, p = 0.001 |
| Scirè, 2009 [47] | 43 | Early RA patients treated with cs-DMARDs in clinical remission (defined as DAS < 1.6 at two consecutive visits 3 months apart, after ≥12 months of follow-up)  Mean± (SD) disease duration 3.8 (±2.8) months | Prospective cohort study | Toshiba Nemio with a multi-frequency linear array transducer (8–14MHz)  A single experienced operator, unaware of clinical data  Inter-observer reliability: exact agreement of 78 and 93% for the presence/absence of GS synovitis and for the PD signal, with k = 0.7 and 0.8, respectively. Using the semi-quantitative grading system, the exact agreement was 62 and 86% for GS synovitis and for the PD signal, with weighted k = 0.6 and 0.8 and ICC 0.8 and 0.9, respectively. The intraobserver reliability: exact agreement of 86 and 98%, with k = 0.7 and 0.9 for the presence/absence of GS synovitis and for the PD signal, respectively. Using the semi-quantitative grading system, exact agreement was 82 and 96%, with weighted k = 0.8 and 0.9 and ICC 0.9, both for GS synovitis and PD signal, respectively  US on 44 joints (bilateral shoulder, elbow, wrist, MCP, PIP, sternoclavicular and acromioclavicular joint, knee, ankle and MTP joints)  GS (US-JC) synovitis and PD (US-PD) were according to the OMERACT definitions | SJC | ***Relapse***  (DAS ≥1.6 following a period of clinical remission)  SJC>1: OR (95% CI) 0.6 (0.1,5-5), p>0.05  US-JC>2: OR (95% CI) 4.6 (0.4,49.5), p>0.05  US-PD>0: OR (95% CI) 12.8 (1.6,103.5), p<0.05 |
| Ten Cate, 2018 [24] | 174 | Early RA, cs-DMARD naive  Median (IQR) symptom duration 46 (2.8-7.9) months | Prospective cohort study  The analysis with imputed data yielded almost equal results | ESAOTE MyLab60, linear array 6–18 MHz  Seven experienced ultrasonographers blinded to clinical findings  Interobserver reliability: ICC 0.58 (95% CI 0.45–0.72) for GSUS, and 0.48 (0.34–0.64) for PDUS, while it was 0.58 (0.49–0.68) for the two modalities combined  Bilateral MCP 2-5, wrists, MTP 2-5  GS, PD and erosions 0-3 | DAS28 | ***Response - 1 yr***  (Defined as absence of DAS28 remission)  DAS 28 per point  OR (95% CI) 1.34 (1.03,1.73)  GS per point  OR (95% CI) 0.99 (0.96,1.02)  PD per point  OR (95% CI) 0.99 (0.95,1.03) |

Summary of findings of articles included in question A1. RA: rheumatoid arthritis; US: ultrasonography; GS: grey-scale; PD: power Doppler; USJCAS: ; USJPD: ultrasonographic joint power Doppler; USJC: ultrasonographic joint count; CF: color fraction; TJC: tender joint count; SJC: swollen joint count; DAS28: disease activity score on 28 joints; HAQ: Health Assessment Questionnaire; MCP: metacarpophalangeal joints; PIP: proximal interphalangeal joints; MTP: metatarsophalangeal joints; RAI: Ritchie articular index; DMARDs: disease modifying antirheumatic drugs; TNFis: TNF inhibitors; ABT: abatacept; OR: odds ratio; RR: risk ratio; sd: standard deviation; IQR: interquartile range; SE: standard error; CI: confidence interval.

Supplementary Table S6: In patients with RA, what is the diagnostic value of ultrasound for active inflammation as compared to clinical examination, using other imaging as reference standard?

| **Study** | **N** | **Population** | **Study design** | **Intervention (US technique)** | **Reference standard** | **Results** |
| --- | --- | --- | --- | --- | --- | --- |
| Abdelzaher, 2019 [51] | 30 | RA with shoulder pain | Cross-sectional study | Toshiba Xario 200 machine with 13 MHz superficial probe  An experienced radiologist with more than 10 years of experience blinded to clinical data and MRI findings of the patients  Shoulders: rotator cuff tendon, long head of the biceps tendon, acromion-clavear joint, subacromiondeltoid bursa | MRI 1.5 T | ***LHB tenosynovitis***  Se 0.87  Sp 0.98  PPV 0.93  NPV 0.95  ***SAD bursitis***  Se 0.72  Sp 0.95  PPCV 0.80  NPV 0.94  ***Subscapularis tenosynovitis***  Se 0.92  Sp 0.98  PPCV 0.92  NPV 0.98 |
| Damjanov, 2012 [52] | 90 | Longstanding RA with active disease | Prospective cohort study | GE Logiq9, 13MHz linear probe  Two independent examiners blinded to clinical findings  Interobserver agreement on ultrasonographic GS synovitis: k values between 0.51 and 0.92 in different joints, for PD assessment k values between 0.74 and 0.78 in different joints  MCP joints, wrists  GS (synovial effusion/hypertrophy) | MRI | ***AUC (95%CI)***  ***US synovitis/effusion wrists*** 0.75 (0.43,1)  ***US synovitis/effusion MCP*** 0.65 (0.53,0.76)  ***Clinical synovitis/effusion wrist*** 0.36 (0.04,0.68)  ***Clinical synovitis/effusion MCP*** 0.55 (0.43,0.66) |
| El-Melegy, 2017 [53] | 20 | Longstanding RA  Mean (sd) disease duration 11.5 (7.8) years | Cross-sectional study | SAMSUNG MEDISON (UGEO H60)  Radiologist and rheumatologist experienced in musculoskeletal imaging  TMJ joint: effusion, erosions, anterior capsule–condyle distances | MRI | US abnormalities were less frequent than MRI abnormalities (77.5% vs 82.5), in particular regarding erosions. US assessment was positive in 77.5% of cases, while clinical examination in 67.5% |
| Ogishima, 2014 [54] | 77 | Longstanding RA treated with cs-DMARDs and/or b-DMARDs  Mean (± SD) disease duration 7.2 (± 5.5) years | Prospective cohort study | Toshiba Aplio TM XG with a 7–14 MHz linear array transducer  US examinations were performed by five rheumatologists not completely blinded to the results of clinical examination, the grades were assigned by consensus among the five rheumatologists  US on 20 joints (MCP, PIP, wrists)  GS graded 0-3 and PD graded 0-3. | MRI  Low-ﬁeld extremity MRI technique, 0.3 T static magnetic ﬁeld  Joint synovitis was deﬁned as synovium showing a high-intensity signal on short tau inversion recovery (STIR) sequence in cMRI | ***MRI synovitis***  US:  Se (95% CI) 0.48 (0.42,0.54)  Sp (95% CI) 0.95 (0.92,0.95) LR+ (95% CI) 7.51 (5.9,9.58)  LR- (95% CI) 0.55 (0.5,0.62)  PPV (95%CI) 0.65 (0.59,0.71)  NPV (95%CI) 0.88 (0.87,0.89)  Clinical examination:  Se (95% CI) 0.43 (0.37,0.49)  Sp (95% CI) 0.87 (0.84,0.89) LR+ (95% CI) 3.29 (2.71,3.99)  LR- (95% CI) 0.66 (0.59,0.72)  PPV (95%CI) 0.45 (0.41,0.50)  NPV (95%CI) 0.86 (0.85,0.87) |
| Taniguchi, 2014 [55] | 30 | Longstanding RA  Mean (sd) disease duration 12.5 (11.5) years | Cross-sectional study | Hitachi HI VISION Avius with a linear probe (14–6 MHz)  A trained orthopedic surgeon performed the ultrasonographic examination, two other orthopedic surgeons specializing in RA scored the joints independently, with no reference to any other clinical information  Interobserver agreement was excellent for MRI scores and PD image scores, κ value ≥0.81  Wrist and MCP | MRI | US vs MRI  *Wrist*  Se (95% CI) 0.64 (0.49,0.77)  Sp (95% CI) 1 (0.73,1)  LR- (95% CI) 0.35 (0.24,0.52)  PPV (95% CI) 1  NPV (95% CI) 0.41 (0.32,0.50)  *MCP*  Se (95% CI) 0.27 (0.18,0.38)  Sp (95% CI) 1 (0.98,1)  LR- (95% CI) 0.73 (0.64,0.83)  PPV (95% CI) 1  NPV (95% CI) 0.77 (0.75,0.80)  Clinical assessment vs MRI  *Wrist*  Se (95% CI) 0.52 (0.37,0.66)  Sp (95% CI) 0 (0,0.26)  LR+ (95% CI) 0.52 (0.4,0.68)  PPV (95% CI) 0.67 (0.61,0.73)  *MCP*  Se (95% CI) 0.31 (0.21,0.41)  Sp (95% CI) 0.98 (0.95,0.99)  LR+ (95% CI) 16.71 (6.01,46.45)  LR- (95% CI) 0.71 (0.61,0.81)  PPV (95% CI) 0.86 (0.70,0.94)  NPV (95% CI) 0.78 (0.75,0.80) |
| Zou, 2020 [56] | 39 | Early RA  Mean (sd) symptom duration 12.2 (10.9) months | Cross-sectional study | ESAOTE MyLab Twice, 12–18MHz linear probe  The same rheumatologist who performed clinical examination performed the ultrasonographic examination and graded it at the point-of-care. To examine inter-rater reliability a second rheumatologist trained in ultrasound retrospectively graded all images blinded to the other rheumatologist’s scores  Inter-rater reliability was excellent for both synovial thickening (k = 0.81) and PD (k = 0.87), and good for erosion (k = 0.65)  Bilateral MTP 2–5  GS and PD 0-3, Pathological GS defined as ≥2 | MRI of the most symptomatic foot | GS  Se (95% CI) 0.59 (0.47,0.70)  Sp (95% CI) 0.35 (0.24,0.46)  LR+ (95% CI) 0.91 (0.71,1.17)  LR- (95% CI) 1.17 (0.78,1.74)  PPV (95% CI) 0.46 (0.40,0.52)  NPV (95% CI) 0.47 (0.37,0.57)  PD  Se (95% CI) 0.69 (0.48,0.85)  Sp (95% CI) 0.58(0.49,0.67)  LR+ (95% CI) 1.67 (1.20,2.31)  LR- (95% CI) 0.53 (0.29,0.95)  PPV (95% CI) 0.25 (0.19,0.31)  NPV (95% CI) 0.90 (0.83,0.94)  Clinical assessment  Se (95% CI) 0.28 (0.18,0.39)  Sp (95% CI) 0.90 (0.82,0.95)  LR+ (95% CI) 2.83 (1.42,5.65)  LR- (95% CI) 0.80 (0.68,0.93)  PPV (95% CI) 0.67 (0.51,0.80)  NPV (95% CI) 0.62 (0.59,0.66) |

Summary of findings of articles included in question A2. RA: rheumatoid arthritis; US: ultrasonography; MRI: magnetic resonance imaging; GS: grey-scale; PD: power Doppler; MTP: metatarsophalangeal joints; LHB: long head of the biceps; SAD: subacromiondeltoid; TMJ: temporo-mandibular joints; cs-DMARDs: conventional synthetic disease modifying antirheumatic drugs; sd: standard deviation; IQR: interquartile range; CI: confidence interval; Se: sensitivity; Sp: specificity; LR+: positive likelihood ratio; LR- negative likelihood ratio; PPV: positive predictive value; NPV: negative predictive value.

Research area B

Supplementary Table S7: In patients with RA, what is the diagnostic value of different ultrasound scores (A vs. B) to detect inflammation using extensive ultrasound assessment as reference standard?

| **Study** | **N** | **Population** | **Study design** | **Intervention (US technique)** | **Comparator** | **Results** |
| --- | --- | --- | --- | --- | --- | --- |
| Dougados, 2009 [4] | 76 | RA starting TNFis, 10±9 years | Prospective cohort study | ESAOTE Technos MPX, Toshiba Aplio, ESAOTE MyLab, Philips HD11, BK Mini Focus  A single sonographer per center (a radiologist or a rheumatologist) with experience (at least 70 different examinations) in evaluating synovitis was in charge of the monthly monitoring of the patients.  Intraobserver reliability (discrimination) was evaluated on the data obtained at screening and baseline visits (range from 0.53 to 0.97).  Scores: 38-, 28- and 20-joints US score  PIP, MCP, elbows, wrists, shoulders, knees, MTPs.  GS and PD | 38-joints US score: the 28 joints included in DAS28 and metatarsophalangeals (MTPs) × 10. | Construct validity measured through Cronbach α  *20-joints US score*  Joint count GS 0.76  Joint count PD 0.81  Joint score GS 0.83  Joint score PD 0.82  *28-joints US score*  Joint count GS mode 0.87 Joint count PD 0.86  Joint score GS 0.89  Joint score PD 0.84  *38-joints US score*  Joint count GS 0.86  Joint count PD 0.85  Joint score GS mode 0.88 Joint score PD 0.85 |
| El-Gohary, 2019 [5] | 50 | Longstanding RA on csDMARDs/bDMARDs  Mean (sd) symptom duration 89.12 (94.05) months | Cross-sectional study | GE LOGIQ P6  Ultrasonography examination was performed, on the same day of clinical and laboratory assessment, by one well-trained rheumatologist with 7-year experience who was blinded to clinical data.  The interobserver / intraobserver reliability was assessed by rereading of selected stored images of 40 patients using randomization technique (significant agreement with p<0.001). κ Values were 0.80 and 0.96 for GS and PD interobserver reliability and 0.96 and 1.0 for GS and PD intraobserver reliability.  7-joints score and 12-joints score  wrist, second and third MCPs, second and third PIPs, elbow, knee, ankle, and second and fifth MTP  GS and PD 0-3  Overall scores for GS and PD as sum | Simplified 12-joint score: bilateral wrist,  2-3 MCP, elbow, knee, ankle.  GS-US12 range 0-72, and PD-US12 0-72 | Spearman’s correlation  GS R 0.690 (P <0.001)  PD R 0.693 (P <0.001). |
| Fukae, 2009 [9] | 69 | RA | Case-control study | Hitachi EUB-6500  Intraobserver reliability was assessed by examining four patients with active RA. The PD-US examinations were repeated ten times for each joint by a single ultrasonographer. Intraobserver reliability was estimated using calculations of intraclass correlation coefficients (ICC). Interobserver reliability was assessed by 31 recording images of the randomly chosen 30 patients with active RA (ICC). The ICC values were 0.97 and 0.99 for intra- and interobserver reliability, respectively.  MCP, PIP joints  quantitative PD assessment vs semiquantitative PD score  PD – quantitative assessment | Semiquantitative PD score: conventional scoring system for finger joint synovial vascularity (0 = absence of signal, 1 = single vessel dots, 2 = vessel dots over less than half area of the synovium, 3 = vessel dots over more than half the area of the synovium). | Regression analysis demonstrated a strong correlation between the scores (R2 = 0.8629, y = 0.02927x + 0.6450). |
| Hammer, 2011 [6] | 20 | Longstanding RA starting ADA  Median (IQR) Disease duration 7.5 (1 - 26) years | Prospective cohort study  No missing examinations during the study. | Siemens Antares  Reliability tests for US scoring of joints were performed on acquired images with median (range) intraobserver intraclass correlation coefficients (95% CI) of 0.97 (0.96 to 0.98) for BM scores and 0.98 (0.97 to 0.99) for PD scores. No reliability tests were, however, performed for the scoring of tendons and bursae.  7-, 12-, 28, 44-joints US score  PIP (10), MCP (10), carpometacarpal (10), wrists, elbows, shoulders, hips, knees, ankles,  4 major foot joints (talonavicular, subtalar, calcaneocuboidal  and cuneonavicular joints), MTP (10),  IP joint of the first toe.  GS and PD, tenosynovitis | Assessment of 78 joints, 36 tendons or tendon groups and 2 bursae (the 78-joint score). Sum scores of GS or PD obtained as the sum of single joints | Spearman’s correlation coefficient at baseline and 12-months  GS 7-joint vs 78-joints score 0.87/0.93  PD 7-joint vs 78-joints score 0.95/0.95  GS 12-joint vs 78-joints score 0.87/0.96  PD 12-joint vs 78-joints score 0.86/0.89  GS 28-joint vs 78-joints score 0.95/0.93  PD 28-joint vs 78-joints score 0.93/0.95  GS 44-joint vs 78-joints score 0.97/0.99  PD 44-joint vs 78-joints score 0.95/0.98 |
| Kawahara, 2019 [11] | 389 | Longstanding RA | Cross-sectional cohort study  Among 10,824 assessed joints, US was not performed on 170 from 27 patients because of severe flexion contracture of the fingers, and, thus, these joints were excluded from the analysis at the joint level. | Toshiba Aplio 400 linear array probe 12 MHz  Each examination and scoring were performed by at least two sonographers from a specially trained team composed of clinical laboratory technologists and rheumatologists.  GS and PD 0-3  PD assessed by SMI  Joints scored as GS ≥ 2 and/or PD ≥ 1 were considered to be inflammatory joints  Patient-oriented US score (PtUS): sum of the US scores of joints in which the patient declared arthralgia  Physician-oriented US score (PhUS): sum of US scores based on joints that each attending physician judged to be a tender joint, swollen joint, or both.  US5: sum of the US scores of unilateral 2MCP, 3MCP, 2PIP, 3PIP, and the wrist. | US22: Bilateral (MCP) 1-5, (PIP/IP) 1-5, and wrist joints | Correlation between patient or physician-oriented US (PtUS or PhUS) and comprehensive US (US22): PtUS showed a moderate correlation (ρ = 0.435, P < 0.001), which was better than PhUS (ρ = 0.383, P < 0.001).  The correlation between US5 and US22 was strong (ρ = 0.813, P < 0.001). |
| Kawashiri, 2011 [7] | 22 | Longstanding RA | Cross-sectional cohort study | TOSHIBA AplioXG, multifrequency linear transducer (12 MHz)  US assessment performed by one Japan College of Rheumatology-certified rheumatologist.  Images from all the examinations were stored, and the US scoring reliability was examined by assessing 24 synovial sites in randomly selected patients at the end of the study.  6 joints: wrists, MCP 2-3  PD graded 0-3 | 12 joints: bilateral elbows, wrists, 2-3 MCP joints, knees, and ankles | The 6j-PDUS scores were strongly correlated with 12j-PDUS scores (r = 0.92, P<0.0001) |
| Naredo, 2005 [8] | 94 | Longstanding RA patients treated with cs-DMARDs and/or b-DMARDs.  Median disease duration (±SD) 69.3 (±58.2) (range 5-280) months. | Cross-sectional cohort study | GE Logiq 400CL (1), GE Logiq 700 (2), 69 patients were examined with scanner 1 and 25 with scanner 2.  A single rheumatologist, experienced in US blinded to the clinical findings performed the US evaluation.  6-10-12(x2)-16-18 joint; effusion and synovitis graded from 0-3 | 60 joint US assessment: (glenohumeral, acromioclavicular, sternoclavicular, elbow, wrist, MCP, and PIP of hands, hips, knees, tibiotalar, subtalar, midtarsal, MTP, and PIP of feet) | ***Pearson and Spearman’s correlation with ultrasonographic effusion joint count (USJCE) 60***  USJCE 6 0.77  USJCE 10 0.82  USJCE 12A 0.83  USJCE 12B 0.88  USJCE 16 0.90  USJCE 18 0.92 |
| Naredo, 2008 [1] | 160 | Longstanding RA patients who started treatment with b-DMARDs.  Mean (sd) disease duration 111.9 (88.6) months (range 4-552 months). | Prospective cohort study | GE Logiq 5 PRO; multifrequency linear array transducers (7–12 MHz)  Rheumatologists experienced in US (1 rheumatologist in 15 centers, 2 rheumatologists in 2 centers, and 3 rheumatologists in 1 center) who were blinded to the clinical and laboratory findings performed US.  Interobserver reliability between US investigators was evaluated before patients’ inclusion by scoring for synovitis and PD signal in 20 recorded images of the joints included in the PDUS assessment from 20 patients with active RA, randomly chosen by the investigator who coordinated the study. Interobserver agreement was significant for both synovitis and PD signal (P<0.0005). Kendall’s W coefficient was 0.5 for synovitis and 0.8 for PD signal.  Reduced 12-joint PDUS assessment (bilateral elbow, wrist, 2-3 MCP, knee, and ankle)  Simpliﬁed 12-joint PDUS model (12S-joint PDUS) total of 24 synovial sites from the 12 selected joints.  Tenosynovitis, and bursitis graded 0-3  Intraarticular, tenosynovial, and intrabursal PD graded 0-3. | Synovitis US count on 44 joints (44-USCS),  PD US count on 44 joints (44-USCPD)  Overall US index for joints with synovitis (44-USIS)  Overall US index for joints with PD signal (44USIPD)  (Sum of synovitis and PD signal scores, respectively, obtained from each joint)  44 joints: bilateral shoulder, elbow, wrist, MCF, PIP of the hands, hip, knee, ankle, tarsal, and MTF joints. | ***Reference 44-USCS/44USIS >0, 44-USCPD/44-USIPD >0***  12-joint PDUS assessment sensitivity of 1 for the 44-USCS/44USIS >0 and 0.94 for the 44-USCPD/44-USIPD >0.  12S-joint PDUS assessment had a sensitivity of 1 for the 44-USCS/44-USIS >0 and 0.91 for the 44-USCPD/44-USIPD >0 |
| Naredo, 2013 [2] | 67 | Longstanding RA patients treated with methotrexate in DAS28 clinical remission.  Mean (±SD) disease duration 7.5 (±5.8) (range 2 to >20) years. | Cross-sectional cohort study | Esaote Mylab 70 XVG, with 6–18MHz transducer for superﬁcial areas, and a 4–13MHz transducer for deep areas  A single rheumatologist experienced in musculoskeletal US, blinded to the clinical, laboratory, and radiographic data, performed US on the day of the clinic visit.  US score A: 20 joint US (wrist, 2-5 MCP, ankle, and 2-5 MTP joint)  US score B: 12-joint US (bilateral elbow, wrist, MCP 2 and 3, knee, and ankles)  Score C: large joints (bilateral glenohumeral, elbow, wrist, hip, knee, and ankle joints)  Score D: wrist and hand (bilateral wrist, MCP 2–5, and PIP joints 2–5)  Score E: reduced wrist and hand (bilateral wrist and MCP joints 2–5)  Score F: 7 joints (wrist, MCP 2 and 3, PIP 2 and 3 of the clinically dominant hand, and MTP joints 2 and 5 of the clinically dominant foot)  Score G: 6 joints (bilateral wrist, MCP 2, and knee joints)  In all scores, synovial hypertrophy and PD graded 0-3. | US of 44-joint  (bilateral glenohumeral, elbow, wrist, 2-5 MCP, 2-5 PIP of the hands, hip, knee, ankle, and 2-5 MTP) | ***Reference 44-US SHI>1, PDI>0***  US score A: sensitivity of 0.97 for SH and 1 for PD  US score B: sensitivity of 0.94 for SH and 0.95 for PD  US score C: sensitivity of 0.78 for SH and 0.21 for PD  US score D: sensitivity of 0.75 for SH and 0.84 for PD  US score E: sensitivity of 0.72 for SH and 0.84 for PD  US score F: sensitivity of 0.64 for SH and 0.68 for PD  US score G: sensitivity of 0.83 for SH and 0.89 for PD |
| Picchianti Diamanti, 2018 [3] | 40 | Longstanding RA patients treated with cs-DMARDs, in clinical remission.  Mean (±SD) disease duration 8.1 (± 5.6) years. | Cross-sectional cohort study | GE Logiq E9 machine with a multi-frequency 6-15 MHz linear transducer.  Two rheumatologists experienced in musculoskeletal US, who was blinded to the clinical data, performed the US assessment.  Inter-observer reliability test (κ coefficient >0.7) between the two US investigators was performed during a pre-study meeting.  US score A: 3 joints (both wrists and the II MCP of the dominant hand).  US score B: 4 joints (both wrists, and both II MCP).  GS and PD graded 0-3. | 18 joints: radiocarpal, II-III-IV-V MCP and II-III-IVV MTP joints bilaterally. | ***Reference 18-joint score***  US score A:  Se (95% CI) 0.90 (0.68,0.99)  Sp (95% CI) 1 (0.83,1)  LR- (95% CI) 0.10 (0.03,0.37)  NPV (95% CI) 0.91 (0.73,0.97)  AUC (95% CI) 95% (0.83,0.99)  US score B:  Se (95% CI) 1 (0.83,1)  Sp (95% CI) 1 (0.83,1)  PPV 1  NPV 1  AUC (95% CI) 1 (0.91,1) |
| Sivakumaran, 2019 [12] | 224 | Longstanding RA  Range of disease duration 95-120 months | Cross-sectional study | GE Logiq S8 equipped with 6-15 MHz probe  18 joints (wrist, MCP 2-5, PIP 2-5)  16 joints (MCP 2-5, PIP 2-5)  14 joints (wrist, MCP 2-4, PIP 2-4)  10 joints (wrist, MCP 2-3, PIP 2-3)  8 joints (MCP 2-3, PIP 2-3)  4 joints (wrist, MCP 5)  4 joints (MCP 2-3)  Evaluation of synovial hypertrophy, PD and erosions.  SH grade 1 score= number of joints with SH 1; SH grade 2 score= number of joint s with SH 2; SH grade 3 score= number of joints with SH 3.  PD score: sum of individual PD scores per patients.  Erosion score: total number of erosions per patients.  Tendons reported separately. | 22 joint assessment (wrist, MCP, PIP) and extensor tendons | Comparable SH and PD scores across different scores, without significant differences.  More limited scores underestimate erosive damage. |
| Tan, 2018 [10] | 24 | RA starting DMARDs | Prospective cohort study | Philips EPIQ 5G, 5–17 MHz multi-frequency linear probe or GE LOGIQe, 5–13 MHz multi-frequency linear probe.  A single rheumatologist with experience in musculoskeletal ultrasound who was blinded to the findings of the clinical joint assessors acquired and scored the ultrasound images.  Novel methods select up to 7 or 12 of the most affected joints via  (i) baseline ultrasound results alone—the Individualized-Ultrasound (IUS) method, or  (ii) (ii) baseline results from both ultrasonography and clinical joint assessments—the individualized-Composite-Ultrasound (ICUS) method. | Existing scores:  7-joint count (wrist, 2-3 MCP, 2-3 PIP, 2-5 MTP)  12-joint count (bilateral elbows, wrists, 2-3 MCP, knees and ankles). | Mean baseline (95%CI) total inflammatory score (TIS), sum of individual joint scores  12 joints  Existing 1.35 (0.97,1.73)  IUS 2.43 (1.78,3.08)  ICUS 2.56 (2.04,3.08)  7 joints  Existing 0.56 (0.38,0.54)  IUS 1.49 (1.46,2.32)  ICUS 1.90 (1.54,2.26) |

Summary of findings of articles included in question B1. RA: rheumatoid arthritis; US: ultrasonography; MRI: magnetic resonance imaging; GS: grey-scale; PD: power Doppler; MTP: metatarsophalangeal joints; LHB: long head of the biceps; SAD: subacromiondeltoid; TMJ: temporo-mandibular joints; cs-DMARDs: conventional synthetic disease modifying antirheumatic drugs; sd: standard deviation; IQR: interquartile range; CI: confidence interval; Se: sensitivity; Sp: specificity; LR+: positive likelihood ratio; LR- negative likelihood ratio; PPV: positive predictive value; NPV: negative predictive value.

Supplementary Table S8. In patients with RA, what is the diagnostic value of different ultrasound scores (A vs. B) to detect inflammation using clinical examination as reference standard?

| **Study** | **N** | **Population** | **Study design** | **Intervention (US technique)** | **Reference standard** | **Results** |
| --- | --- | --- | --- | --- | --- | --- |
| Dougados, 2009 [4] | 76 | Longstanding RA starting TNFis  Mean (sd) disease duration 10 (9) years | Prospective cohort study | ESAOTE Technos MPX, Toshiba Aplio, ESAOTE MyLab, Philips HD11, BK Mini Focus  A single sonographer per center (a radiologist or a rheumatologist) with experience (at least 70 different examinations) in evaluating synovitis was in charge of the monthly monitoring of the patients.  Intraobserver reliability (discrimination) was evaluated on the data obtained at screening and baseline visits (range from 0.53 to 0.97).  38-, 28- and 20-joints US score  PIP, MCP, elbows, wrists, shoulders, knees, MTP joints  Lesions: GS and PD | Clinical examination | Construct validity measured through Cronbach α  *20-joints US score*  Joint count GS 0.76  Joint count PD 0.81  Joint score GS 0.83  Joint score PD 0.82  *28-joints US score*  Joint count GS mode 0.87 Joint count PD 0.86  Joint score GS 0.89  Joint score PD 0.84  *38-joints US score*  Joint count GS 0.86  Joint count PD 0.85  Joint score GS mode 0.88 Joint score PD 0.85 |
| El-Gohary, 2019 [5] | 50 | Longstanding RA on csDMARDs/bDMARDs  Mean (sd) symptom duration 89.12 (94.05) months | Cross-sectional study | GE LOGIQ P6  Ultrasonography examination was performed, on the same day of clinical and laboratory assessment, by one well-trained rheumatologist with 7-year experience who was blinded to clinical data.  The interobserver / intraobserver reliability was assessed by rereading of selected stored images of 40 patients using randomization technique (significant agreement with p<0.001). κ Values were 0.80 and 0.96 for GS and PD interobserver reliability and 0.96 and 1.0 for GS and PD intraobserver reliability.  7-joints score and 12-joints score  wrist, second and third MCPs, second and third PIPs, elbow,  knee, ankle, and second and fifth MTP  GS and PD 0-3  Overall scores for GS and PD as sum | Clinical examination | Spearman’s correlation  between 12-joints score and TJC/SJC, respectively:  GS R 0.115, 0.341 (p=0.428, 0.015)  PD R 0.100, 0.418 (p=0.488, 0.003).  Spearman’s correlation  between 12-joints score and TJC/SJC, respectively:  GS -0.068, 0.242 (p=0.637, 0.090)  PD R 0.121, 0.396 (p =0.401, 0.004). |
| Kawashiri, 2011 [7] | 22 | Longstanding RA | Cross-sectional cohort study | TOSHIBA AplioXG, multifrequency linear transducer (12 MHz)  US assessment performed by one Japan College of Rheumatology-certified rheumatologist.  Images from all the examinations were stored, and the US scoring reliability was examined by assessing 24 synovial sites in randomly selected patients at the end of the study.  6 joints: wrists, MCP 2-3  12 joints: bilateral elbows, wrists, 2-3 MCP joints, knees and ankles  PD graded 0-3 | Clinical examination | Spearman’s correlation  12j-US/DAS28 0.72 p<0.001  12j-US/SDAI 0.6 p=0.006  6j PDUS/DAS-28 0.67 P<0.01  6j PDUS/SDAI 0.55 p<0.05 |

Summary of findings of articles included in question B2. RA: rheumatoid arthritis; US: ultrasonography; GS: grey-scale; PD: power Doppler; MTP: metatarsophalangeal joints; sd: standard deviation; DAS28: disease activity score on 28 joints; TNFis: tumor necrosis factor inhibitors.

Supplementary Table S9: In patients with RA, what is the value of different ultrasound scores to predict outcome?

| **Study** | **N** | **Population** | **Study design** | **Intervention (US technique)** | **Comparator** | **Results** |
| --- | --- | --- | --- | --- | --- | --- |
| Ellegaard, 2014 [13] | 46 | Longstanding RA starting TNFis  Median (IQR) disease duration 6.5 (3.5-13) years | Prospective cohort study | Siemens  4 experienced ultrasonographers performed US evaluation.  Wrists  Quantitative scoring of colour fraction of PD signal + Semiquantitative scoring (SQS) of PD signal.  PD | Semiquantitative scoring (SQS) of PD signal: grade 0: no PD signal; grade 1: up to 2 single PD spots; grade 2: more than grade 1 and up-to 50% PD activity in the joint; grade 3: more than 50% PD activity in the joint. | ***Response – 1 yr***  The discriminative ability of both scores (SQS and QS) to predict treatment success at 1 year measured as decrease in DAS28 was poor. |
| Fukae, 2010 [14] | 19 | Early RA starting cs-DMARDs  Median (IQR) symptom duration 5 (3–11) months | Prospective cohort study | Hitachi EUP-L34P, EUB-7500, Hitachi  US was performed by 3 ultrasonographers specialized in musculoskeletal ultrasonography who were blinded to other clinical information.  All PDS images for MCP joints and PIP joints were blindly evaluated twice according to the semiquantitative score for each joint by 2 ultrasonographers. The intraobserver kappa values of the semiquantitative score were 0.944 for MCP joints and 0.930 for PIP joints. The intraobserver overall agreement for these joints was 96% and 95.4%, respectively. The obtained interobserver kappa values of the semiquantitative score were 0.950 for MCP joints and 0.923 for PIP joints. The interobserver overall agreement for these joints was 95.7% and 97.1%, respectively.  MCP and PIP  Quantitative assessment of PD determined by counting the number of vascular flow pixels in the region of interest (ROI)  Semiquantitative score for PD 0-3 | Semiquantitative score for PD | ***Radiographic progression – 20 weeks***  (Genant modified Sharp score)  Baseline quantitative PD correlated with local progression in MCP and PIP (Spearman’s ρ= 0.466, p 0.0001 and Spearman’s ρ= 0.362, p < 0.0001, respectively). The correlation between the semiquantitative score and local progression had the same tendency.  The level of quantitative PD improvement between W8 and baseline negatively correlated with local progression in MCP (Spearman’s ρ =  -0.340, p=0.00386), but not in PIP (Spearman’s ρ =-0.223, p= 0.1430). The level of semiquantitative score improvement between W8 and baseline and the local progression were not correlated at MCP and PIP (Spearman’s ρ=-0.256, p =0.0579 and Spearman’s ρ=-0.105, p = 0.5179, respectively). |
| Janta, 2016 [15] | 47 | Longstanding RA in clinical remission  Mean (sd) disease duration 9.4(6.9) years | Prospective cohort study | ESAOTE Mylab 70 6-18-MHz and 4-13 MHz transducer  One rheumatologist experienced in US performed US assessment, blinded to clinical data.  GS and PD 0-3 for synovitis and tenosynovitis.  large joints (bilateral glenohumeral, elbow, wrist, hip, knee, and ankle)  wrist and hand (wrist, MCP 2-5, PIP 2-5)  reduced wrist and hand (wrist, MCP 2-5)  wrist– MCP–ankle–MTP (wrist, MCP 2-5, ankle, MTP 2-5)  12-joints (elbow, wrist, 2-3 MCP, knee and ankle)  7-joints (wrist, 2-3 MCP, 2-3 PIP of the clinically dominant hand, 2-5 MTP of the clinically dominant foot)  6-joints (wrist. MCP 2, knee) | 44 J glenohumeral, elbow, wrist, MCP 2-5, PIP 2-5, hip ,knee, ankle, MTP 2-5. 2-6 wrist extensor compartments, 2-5 finger flexor digitorum superficialis and profundus tendons, and tibialis posterior tendon. | ***Relapse – 6 months***  (Unstable remission defined as DAS28 ≥ 2.6 having had no changes in RA therapy or as an increase in RA therapy because of disease relapse)  PD in 44 joints  OR (95%CI) 8.21(1.49,45.41)  44-joint PDindex  OR (95%CI) 2.20 (1.01,4.28)  SH in wrist-MCP  OR (95%CI) 4.79(1.04,22.17)  PD in wrist-MCP-ankle-MTP  OR (95%CI) 4.62 (1.03,20.74)  PD in 12 j  OR (95%CI) 5.82 (1.07,31.61)  12 joint PD index  OR (95%CI) 4.19 (1.26,13.96) |

Summary of findings of articles included in question B3. RA: rheumatoid arthritis; PD: power Doppler; SQS: semiquantitative score; QS: quantitative score; MCP: metacarpophalangeal; PIP: proximal interphalangeal joint; IQR: interquartile range; W8: week 8.

Supplementary Table S10.  Studies evaluating the value of different ultrasound scores (A vs. B) to predict outcome. Outcomes of interest are reported in the grey boxes.

| **Study** | **Number of patients** | **Population** | **Intervention (US technique A)** | **Comparator (US technique B)** | **Discriminative ability of US score A vs B to predict outcome** |
| --- | --- | --- | --- | --- | --- |
| **Clinical disease activity** | | | | | |
| Ellegaard, 2014 [13] | 46 | Longstanding RA  Median disease duration: 6.5 years  Follow-up: 12 months  Treatment: TNFis | Quantitative scoring of colour fraction of PD signal.  Joint: wrists | Semiquantitative scoring of PD signal (0-3).  Joint: wrists | A = B  Poor discriminative ability for both scores in predicting treatment success. |
| **Radiographic progression** | | | | | |
| Fukae, 2010 [14] | 19 | Early RA  Median symptoms duration: 5 months  Follow-up: 20 weeks  Treatment: cs-DMARDs | Quantitative assessment of PD.  Joints: MCP and PIP joints. | Semiquantitative scoring of PD signal (0-3).  Joints: MCP and PIP joints. | A = B  Baseline PD scores correlated with outcome.  A > B  Longitudinal improvement in quantitative PD at MCP negatively correlated with radiographic progression at MCP, but not at PIP joints.  Longitudinal variation in semiquantitative PD did not correlate with outcome. |
| **Clinical relapse** | | | | | |
| Janta, 2016 [15] | 47 | Longstanding RA in clinical remission  Mean (SD) disease duration: 9.4 years  Follow-up: 6 months  Treatment: MTX | 12-joints score  Joints: wrist, MCP joints score | 44 joints score  Joints: gleno-humeral, elbow, wrist, MCP 2-5, PIP 2-5, hip, knee, ankle, MTP 2-5.  2-6 wrist extensor compartments, 2-5 finger flexor digitorum superficialis and profundus tendons, and tibialis posterior tendon. | A = B  Both scores predicted unstable remission under MTX therapy. |

Abbreviations: RA: rheumatoid arthritis; US: ultrasound; PD: power Doppler; TNFis: tumor necrosis factor inhibitors; MCP: metacarpophalangeal; PIP: proximal interphalangeal; MTX: methotrexate; DMARDs: disease-modifying anti-rheumatic drugs

Research area C

Supplementary Table S11. In patients with RA, what is the value of ultrasound lesion A versus ultrasound lesion B to predict outcome?

| **Study** | **N** | **Population** | **Study design** | **Intervention (US technique)** | **Comparator** | **Results** |
| --- | --- | --- | --- | --- | --- | --- |
| Chen, 2017 [26] | 32 | Longstanding RA in TNFis | Prospective cohort study | ESAOTE MyLab70, 6-18 MHz transducer  At each centre, a sonographer performed ultrasound examination, an experienced observer blinded to all other study findings scored the images in random order.  Intraobserver reliability: weighted κ values were median 0.8 for GS synovitis and 0.6 for PDUS.  12 joints (bilateral elbows, wrists, MCP 2-3, PIP 2-3)  GS and PD graded 0-3 | DAS28 | ***Radiographic progression – 1 yr***  Multiple linear regression  DAS28 coefficient 0.13, SE 0.35, p 0.719  No improvement in GS 0-1 month coefficient 0.50, SE 0.22, p 0.036  No improvement in GS 0-3 months coefficient 0.04, SE 0.70, p 0.487  No improvement in PD 0-1 month coefficient 0.04, SE 0.36, p 0.653  No improvement in PD 0-3 months coefficient 0.04, SE 0.29, SE 0.36, p 0.250 |
| Filippou, 2018 [31] | 340 | Longstanding RA in clinical remission  Mean (sd) 9.75 (8.07) years | Prospective cohort study  Missing data were handled using available case analysis; in each analysis, all cases with available data on the relevant variables were included. | ESAOTE MyLab 70XVG, MyLab Twice, Logiq9, LogiqE9  Ultrasonographers were rheumatologists expert in MSUS selected by an inter-observer and intra-observer reliability exercise against a reference standard (AI) on static images using an e-learning platform. A good to excellent reliability (weighted kappa ≥0.7) was required.  22 joints (wrists, MCPs, PIPs) + 22 tendons  Lesions: synovial GS and PD 0-3 | GS and PD tenosynovitis | ***Disease flare – 1 yr***  PD + synovitis:  Adj OR (95%CI) 1.59 (0.86,2.92)  GS+ synovitis:  Adj OR (95%CI) 1.88 (0.7,4.46)  PD+ tenosynovitis:  Adj OR (95%CI) 0.47 (0.12,1.82)  GS+ tenosynovitis:  Adj OR (95%CI) 1.37 (0.42,4.41)  ***Radiographic progression – 1 yr***  PD + synovitis:  Adj OR (95%CI) 1.6 (0.68-3.79)  GS+ synovitis:  Adj OR (95%CI) 1.78 (0.49-6.48)  PD+ tenosynovitis:  Adj OR (95%CI) 0.54 (0.05,5.76)  GS+ tenosynovitis:  Adj OR (95%CI) 0.67 (0.06-7.5) |
| Funck-Brentano, 2013 [57] | 127 | Early DMARD-naïve RA  Mean (sd) symptom duration 102.6 (53) months | Prospective cohort study | Toshiba Aplio instrument, ESAOTE Technos MPX  For each center, one trained examiner who was a radiologist or a rheumatologist, blinded to clinical data, performed US.  The examiners agreed on definitions of synovitis and bone erosions before the beginning of the study.  MCP (2-5), V MTP joints  GS, PD, erosions 0-3 | US erosions | ***Radiographic progression – 1 yr***  US erosions:  OR (95% CI) 4.26 (2.39,7.59)  GS:  OR (95% CI) 1.51 (1.01,2.28)  PD:  OR (95% CI) 1.97 (1.26–3.07) |
| Geng, 2016 [34] | 126 | Longstanding RA in clinical remission  Mean (sd) disease duration 5.2 (6.1) years | Prospective cohort study | Esaote Mylab  Two experienced rheumatologists blinded to all clinical findings performed US.  Bilateral wrists, MCPs and PIPs  GS, PD, tenosynovitis, erosions 0-3 | GS synovitis | ***Disease flare***  Baseline PD score:  OR (95% CI) 1.4 (0.9,2.0)  Baseline GS score:  OR (95% CI) 0.7 (0.5, 1.0) |
| Harman, 2018 [35] | 48 | Early RA  Mean (sd) symptom duration 3.12 (1.33) months | Prospective cohort study | GE LOGIQ P5  A trained ultrasonographer with 4 years of experience performed US.  28 joints (MCP (10), PIP (10), wrist, elbow, shoulder, knee joints)  28 joints: MCP joints, wrists, elbows, knees, ankles; MTP  PD synovitis | GS synovitis | ***Radiographic progression – 18 months***  Baseline total GS synovitis scores B:0.091 beta:0.417 p=0.011  1-month total GS synovitis scores B:0.518 beta:0.549 p=0.028  3-months total GS synovitis scores B:2.261 beta:1.110 p=0.278  Baseline total PDUS synovitis scores B:0.057 beta:0.476 p=0.015  1-month total PDUS synovitis scores B:0.075 beta:0.358 p=0.017  3-months total PDUS synovitis scores B:1.154 beta:0.214 p=0.507 |
| Harman, 2015 [36] | 68 | Early RA,  Mean (sd) symptom duration 0.63 (0.61) years | Prospective cohort study  Available data from four patients with incomplete follow-up were excluded from the study. | GE LOGIQ P5  Two trained ultrasonographers with 1 and 3 years of experience performed US.  Interobserver agreement was 87, 91, 77, and 92 % for the presence/absence of GSUS synovitis, PDUS synovitis, GSUS tenosynovitis, and PDUS tenosynovitis with κ values of 0.70, 0.72, 0.64, and 0.78, respectively.  28 joints (MCP joints, wrists, elbows, knees, ankles; MTP)  GS, PD, tenosynovitis, erosions 0-3 | GS synovitis | ***Response – 1 year***  (Defined as DAS at 1 year  Correlation between time-integrated value of clinical and US parameters and disease DAS44  Baseline TJC: 0.35 (p<0.05)  Baseline SJC: 0.31 (p<0.05)  Baseline DAS44: 0.79 (p<0.001)  Baseline total GSUS synovitis scores: 0.38 (p<0.01)  Baseline total PDUS synovitis scores: 0.39 (p<0.01)  Baseline total tenosynovitis scores: 0.36 (p<0.01)  Baseline total PD tenosynovitis scores: 0.41 (p<0.01)  ***Radiographic progession – 1 yr***  (Defined as total radiographic score progression)  Correlation between time-integrated value of clinical and total radiographic score progression  Baseline TJC: 0.33 (p<0.05)  Baseline SJC: 0.35 (p<0.05)  Baseline DAS44: 0.37 (p<0.05)  Baseline total GSUS synovitis scores: 0.54 (p<0.01)  Baseline Total PDUS synovitis scores: 0.56 (p<0.001)  Baseline total tenosynovitis scores: not significant  Baseline total PD tenosynovitis scores: not significant |
| Horton, 2016 [37] | 105 | Early RA on DMARDs treatment  Median (IQR) symptom duration 6 (4-13) months | Prospective cohort study  To determine any difference between patients included in analyses and those excluded due to missing data, Chi-squared tests or Fisher’s exact tests or t-tests for continuous variables were applied. The number of missing values for GS and PD were reported in Table 1. | GE Logiq E9  A validated sonographer who had undergone training with an experienced EULAR teacher and who was blinded to the clinical findings performed US.  26 joints (elbows, wrists, second and third MCP and PIP joints, knees, ankles and MTP joints).  PD synovitis 0-3 | GS synovitis | ***Remission – 1 yr***  (Defined based on DAS28CRP, DAS44CRP, Boolean definition)  *Prediction of DAS28CRP<2.6*  Baseline SJC28 OR (95% CI) 0.98 (0.89, 1.08) NS  Baseline DAS28-CRP OR (95% CI) 0.66 (0.45, 0.97) p=0.04  Total GS score OR (95% CI) 1.00 (0.96, 1.04) NS  Total PD score OR (95% CI) 0.98 (0.92, 1.05) NS  *Prediction of DAS44CRP<1.6*  Baseline SJC28 OR (95% CI) 0.97 (0.87, 1.07) NS  Baseline DAS28CRP OR (95% CI) 0.57 (0.38, 0.86) p=0.008  Total GS score OR (95% CI) 0.98 (0.94, 1.02) NS  Total PD score US OR (95% CI) 0.97 (0.91, 1.04) NS  *Prediction of Boolean remission*  Baseline SJC28 OR (95% CI) 1.00 (0.87, 1.14) NS  Baseline DAS28CRP OR (95% CI) 0.53 (0.30, 0.95) p=0.03  Total GS score OR (95% CI) 1.00 (0.95, 1.05) NS  Total PD score OR (95% CI) 0.98 (0.89, 1.07) NS |
| Ikeda, 2013 [38] | 69 | RA starting MTX or bDMARDs  Median (IQR) disease duration 35 (17–111) months | Prospective cohort study | GE LOGIQ 7 Pro, GE LOGIQ E9, Toshiba Viamo, Hitachi HI VISION Avius  2 rheumatologists trained for musculoskeletal US, who were blinded to clinical information and laboratory data, performed US.  Intraobserver reliability of US assessment was evaluated by randomly selecting 4 images per each joint region from stored images of baseline US examination. Fifty-six images per sonographer were graded again for GS synovitis and synovial PD signal by the same sonographer under a blinded condition at the end of the study period. Interobserver reliability between sonographers was evaluated with the same sets of images. ICC for intraobserver reliability of GS and PD scores were very high (0.93 and 0.97, respectively) and ICC for interobserver reliability of GS and PD scores were also high (0.85 and 0.89).  28 joints (PIP (1–5), MCP (1–5), elbows, wrists, shoulders, knees)  GS and PD 0-3 | GS synovitis | ***Radiographic progression – 24 weeks***  Correlation (Spearman’s correlation coefficient) between cumulative scores (sum of net weights at baseline, 12 weeks, 24 weeks) radiographic progression at 24 weeks.  Cumulative DAS28-CRP: 0.342 (p=0.009).  Cumulative total GS score: 0.062 (p=0.649).  Cumulative total PD score: 0.357 (p=0.006). |
| Inanc, 2016 [39] | 39 | RA starting 1^st^-line TNFis, 9.14 years | Prospective observational study (letter) | Equipment: MyLab70 (Esaote, Italy)  Lesions: GS and PD  Score: semiquantitative scoring system, 28-joints score  28 joints (PIP (1–5), MCP (1–5), elbows, wrists, shoulders, knees) | GS synovitis | ***Response – 3 months***  (Defined as EULAR response)  Baseline PD score in responders (mean (SD)): 13.0 (5.1)  Baseline PD score in non-responders (mean (SD)): 20.6 (11.8); p=0.035  Baseline GS score in responders (mean (SD)): 16.4 (6.9)  Baseline GS score in non-responders (mean (SD)): 24.9 (14.4); p=0.054 |
| Janta, 2016 [15] | 47 | Longstanding RA in clinical remission  Mean (sd) disease duration 9.4(6.9) years | Prospective cohort study | ESAOTE Mylab 70 6-18-MHz and 4-13 MHz transducer  One rheumatologist experienced in US performed US assessment, blinded to clinical data.  GS 0-3 for synovitis and tenosynovitis.  44 J glenohumeral, elbow, wrist, MCP 2-5, PIP 2-5, hip ,knee, ankle, MTP 2-5. 2-6 wrist extensor compartments, 2-5 finger flexor digitorum superficialis and profundus tendons, and tibialis posterior tendon.  large joints (bilateral glenohumeral, elbow, wrist, hip, knee, and ankle)  wrist and hand (wrist, MCP 2-5, PIP 2-5)  reduced wrist and hand (wrist, MCP 2-5)  wrist– MCP–ankle–MTP (wrist, MCP 2-5, ankle, MTP 2-5)  12-joints (elbow, wrist, 2-3 MCP, knee and ankle)  7-joints (wrist, 2-3 MCP, 2-3 PIP of the clinically dominant hand, 2-5 MTP of the clinically dominant foot)  6-joints (wrist. MCP 2, knee) | PD 0-3 for synovitis and tenosynovitis. | ***Relapse – 6 months***  (Unstable remission defined as DAS28 ≥ 2.6 having had no changes in RA therapy or as an increase in RA therapy because of disease relapse)  PD in 44 joints  OR (95%CI) 8.21(1.49,45.41)  44-joint PDindex  OR (95%CI) 2.20 (1.01,4.28)  SH in wrist-MCP  OR (95%CI) 4.79(1.04,22.17)  PD in wrist-MCP-ankle-MTP  OR (95%CI) 4.62 (1.03,20.74)  PD in 12 j  OR (95%CI) 5.82 (1.07,31.61)  12 joint PD index  OR (95%CI) 4.19 (1.26,13.96) |
| Kawashiri, 2017 [41] | 40 | Longstanding RA in clinical remission or LDA, stopping biologics  Mean (sd) disease duration 3.5 (5.5) | Prospective cohort study | Toshiba Aplio 500, Hitachi Ascendus, Hitachi Noblus, GE  Five rheumatologists trained for musculoskeletal ultrasound (Japan College of Rheumatology-certified sonographers) performed US.  LOGIQ E9, multifrequency linear transducer (12–14 MHz)  22 joints (bilateral wrist 1-5 MCP, 1-5 PIP  Bone erosions 0-1 | GS and PD synovitis | ***Relapse – 1 yr***  Only the presence of bone erosion detected by ultrasound at bDMARD discontinuation was an independent predictive variable for relapse  (OR 8.35, 95% CI 1.78–53.2, p = 0.006). Synovitis was not a significant predictor. |
| Nozaki, 2018 [58] | 86 | Longstanding RA starting TNFis  Median (range) disease duration was 6.9 (1–9.7) years | Retrospective cohort study | GE Healthcare Venue 40 with a linear probe at 8–13MHz.  Two rheumatologists blinded to study findings performed US.  The kappa coefficients of inter-observer reliability between the two observers were 0.78 for the SHI values and 0.91 for the PD signal scores (reading of captured images). The intra-observer reliability assessment was good: kappa values 0.90 and 0.88 for the SHI and 0.93 and 0.88 for the PD signal scores.  SH PD assessed in 48 sites in 28 joints, graded 0-3. The sum of the synovial SH scores obtained for each joint region resulted in possible scores of 0–144 for the synovial hypertrophy index (SHI). | PD assessed in 48 sites in 28 joints, graded 0-3. The sum of the synovial PD signal scores obtained for each joint region resulted in possible scores of 0–144 for the Doppler synovitis index (DSI) | ***Clinical response using DAS28-CRP remission criteria***  SHI: OR (95% CI) 6.7 (1.9–27.4)  DSI: OR (95% CI) 3.1 (0.9–12.3)  ***Multivariate analysis***  Logisitc regression using SHI ≤34 and DSI ≤7,  Predictive factors of remission at 54 weeks: a baseline DAS28≤2.7 as low disease activity/remission, and an SHI ≤34 (not DSI) |
| Ramirez, 2017 [59] | 42 | Longstanding RA patients in clinical remission (DAS28 <2.6) for >6 months treated with cs-DMARDs and/or b-DMARDs.  Disease duration, median (inter- quartile range) 93 (55.8–148.2) months. | Prospective cohort study | Siemens Antaress, frequency 8-12MHz.  An experienced sonographer blinded to clinical data performed analyses.  For intra-observer reliability, the ultrasonographer repeated the ultrasound assessment in the first 10 patients included in the study. The two evaluations were separated between 24 and 72 hours. The same sonographer made both ultrasound explorations and noted the results. Intra-rater agreement was 0.81 for SH and 0.92 for PD.  Both knees and 11 joints of each hand (including the PIP joints, MCP joints, and the wrists) for both SH and intra-articular PD signals according to EULAR guidelines.  Patients with SH grade ≥2 plus PD signal were classified as having US-defined active synovitis. | PD alone | ***Radiographic progression***  Patients fulfilling the more stringent US-defined active synovitis criteria (p = 0.039), but not those with PD alone (p = 0.09), had significantly more progression of erosions at 12 months. |
| Saleem, 2012 [45] | 93 | Longstanding RA patients in remission (no flares of disease in the last 6 months, stable treatment with c-DMARDs for 6 months and no indication for a change in treatment).  Median (interquartile range) disease duration 7.0 (4.5–9.5) | Prospective cohort study  Patients with missing data excluded from primary analyses. | Phillips ATL HDI 3000, 10–5 MHz ‘hockey stick’ transducer.  A single experienced ultrasonographer who was blinded to all other study findings performed US.  Satisfactory levels of interobserver and intraobserver reliability data (citations of preliminary works).  The dominant hand MCP joints 2–5 and wrist, dorsal and palmar aspects.  Patients with GS SH grade ≥2 plus PD signal were classified as having US-defined active synovitis. | PD activity scored according to OMERACT definitions. | ***Disease flare***  (Increase in disease activity that required an initiation, change or increase in therapy)  US PD (score >0)  RR (95% CI) 3.0172 (1.12,8.11)  US GS (score >0)  OR (95% CI) 1.44 (0.28,7.32) |
| Saleem, 2010 [60] | 47 (US data in 40) | Longstanding RA patients in clinical remission (DAS28 <2.6)  Median (IQRe) disease duration, months: early treated group: 19 (16-22); delayed treated group: 120 (72-255). | Prospective cohort study  Patients with missing values were given an intermediate score to preserve the number of patients entered into the analysis, allowing the authors to test whether missing data were associated with higher or lower than expected odds of achieving sustained remission. | Phillips ATL HDI 5000, 15-8 MHz ‘hockey stick’ transducer  Dominant hand MCP joints 1 -5, PIP 1-5 and wrist.  GS synovial hypertrophy | PD activity scored according to OMERACT definitions. | ***Disease flare***  (Increase of DAS28 >2.6 or an increase of 1.2 if DAS remained <2.6)  GS synovial hypertrophy:  RR (95% CI) 0.79 (0.47,1.30)  PD activity:  RR (95% CI) 1.10 (0.70,1.73) |
| Scirè, 2009 [47] | 43 | Early RA on cs-DMARDs in clinical remission (DAS < 1.6 at two consecutive visits 3 months apart, after ≥12 months of follow-up)  Mean (±SD) disease duration 3.8 (± 2.8) months | Prospective cohort study | Toshiba Nemio, multi-frequency linear array transducer (8–14MHz).  Experienced operators, unaware of clinical data, performed US.  Inter-observer reliability was evaluated by comparing the findings of the two independent experienced rheumatologist ultrasonographers who performed US examinations of a series of 15 patients. The exact agreement of 78 and 93% for the presence/absence of GS synovitis and for the PD signal, with k ¼ 0.7 and 0.8, respectively.  US assessment of bilateral shoulder, elbow, wrist, MCP, PIP of the hands, sternoclavicular and acromioclavicular joint, knee, ankle and MTP joints.  GS (US-JC) synovitis | PD signal (US-PD) | ***Disease flare***  Defined as a DAS ≥1.6 following a period of clinical remission  US-JC>2: OR (95% CI) 4.6 (0.4,49.5), p>0.05  PD>0: OR (95% CI) 12.8 (1.6,103.5), p<0.05. |
| Tan Cate, 2018 [24] | 174 | Early RA, cs-DMARD naive  Median (IQR) symptom duration 46 (2.8-7.9) months | Prospective cohort study | ESAOTE MyLab60, linear array 6–18 MHz  Seven ultrasonographers, blinded to clinical findings, had been certified for US by the Dutch College of Rheumatology. Prior to the start of the study, after a meeting consensus, an atlas was prepared with sample images and cartoons for each grade of GSUS, PDUS, and erosive changes. This was distributed to all ultrasonographers, and four training sessions prior to and five more during the study were organized to optimise both interpretation and acquisition reliability between ultrasonographers. Interobserver reliability at the joint level between the seven ultrasonographers was 0.58 (95% confidence interval (CI) 0.45–0.72) for GSUS, and 0.48 (0.34–0.64) for PDUS, while it was s 0.58 (0.49–0.68) for the two modalities combined.  Bilateral MCP 2-5, wrists, MTP 2-5  PD 0-3 | GS 0-3 | ***Absence of DAS28 remission – 1 yr***  GS score per point  OR (95% CI) 0.99 (0.96,1.02)  PD score per point  OR (95% CI) 0.99 (0.95,1.03) |

Summary of findings of articles included in question C1. RA: rheumatoid arthritis; US: ultrasonography; GS: grey-scale; PD: power Doppler; SH: synovial hypertrophy; SHI: synovial hypertrophy index; USJC: ultrasonographic joint count; MCP: metacarpophalangeal; PIP: proximal interphalangeal; MTP: metatarsophalangeal joints; cs-DMARDs: conventional synthetic disease modifying antirheumatic drugs; b-DMARDs: biologic disease modifying antirheumatic drugs; TNFis: TNF inhibitors; MTX: methotrexate; TJC: tender joint count; SJC: swollen joint count; DAS: disease activity score; DAS28-CRP: disease activity score on 28 joints based on C reactive protein; sd: standard deviation; IQR: interquartile range; CI: confidence interval; OR: odds ratio; RR: risk ratio.

Supplementary Table S12. What is the diagnostic value of ultrasound lesion A versus lesion B for the diagnosis of active RA as compared to OA?

| **Study** | **N** | **Population** | **Study design** | **Intervention (US technique)** | **Reference standard** | **Results** |
| --- | --- | --- | --- | --- | --- | --- |
| Ehrenstein, 2018 [16] | 43 RA, 57 OA, FM and arthralgia of unknown cause | RA DMARD-naïve  Median (IQR) symptom duration 4.0 (0.5-24.0) months | Prospective cohort study | GE Logiq  7-joints score  Wrists, MCP (2-3), PIP (2-3), MTP (2-5) joints  GS and PD, erosions, tenosynovitis | Clinical diagnosis | *GS synovitis*  Se (95% CI) 0.98 (0.88,0.99)  Sp (95% CI) 0.36 (0.18, 0.57)  LR+ (95% CI) 1.53 (1.13,2.06)  LR- (95% CI) 0.06 (0.01,0.48)  PPV (95% CI) 0.01 (0.01,0.02)  NPV (95% CI) 0.99 (0.99-0.99)  AUC (95% CI) 0.37 (0.25,0.49)  *PD synovitis*  Se (95% CI) 0.74 (0.58,0.86)  Sp (95% CI) 0.84 (0.64,0.95)  LR+ (95% CI) 4.65 (1.86,11.61)  LR- (95% CI) 0.30 (0.18,0.52)  PPV (95% CI) 0.04 (0.01,0.10)  NPV (95% CI) 0.99 (0.99,0.99)  AUC (95% CI) 0.84 (0.73,0.92)  *Erosions*  Se (95% CI) 0.14 (0.05,0.28)  Sp (95% CI) 1 (0.86,1)  LR+ (95% CI) N.A.  LR- (95% CI) 0.86 (0.76,0.97)  PPV 1  NPV (95% CI) 0.99 (0.99,0.99)  AUC (95% CI) 0.99 (0.93,1)  *Tenosynovitis*  Se (95% CI) 0.63 (0.45,0.79)  Sp (95% CI) 0.88 (0.69,0.97)  LR+ (95% CI) 5.30 (1.78,15.81)  LR- (95% CI) 0.41 (0.26,0.66)  PPV (95% CI) 0.05 (0.01,0.14)  NPV (95% CI) 0.99 (0.99,0.99)  AUC (95% CI) 0.88 (0.76,0.95) |
| Fukae, 2009 [9] | 69 | RA | Case-control study | Hitachi EUB-6500  Intraobserver reliability was assessed by examining four patients with active RA. The PD-US examinations were repeated ten times for each joint by a single ultrasonographer. Intraobserver reliability was estimated using calculations of intraclass correlation coefficients (ICC). Interobserver reliability was assessed by 31 recording images of the randomly chosen 30 patients with active RA (ICC). The ICC values were 0.97 and 0.99 for intra- and interobserver reliability, respectively.  20 joints: 20 joints (MCP, PIP joints)  PD Joint synovial vascularity index (JSVI) | Clinical diagnosis | Patients with total JSVI>36% vs JSVI<36%  Se (95% CI) 0.92 (0.75,0.97)  Sp (95% CI) 0.91 (0.73,0.99)  LR+ (95% CI) 11.08 (2.93,41.95)  LR- (95% CI) 0.08 (0.02,0.32)  PPV (95% CI) 0.10 (0.03,0.29)  NPV (95% CI) 0.99 (0.99,0.99) |
| Glimm, 2016 [17] | 90 (67 RA – 23 OA) | Longstanding RA  Mean (sd) symptom duration 7.1 (8.2) years | Cross-sectional study | ESAOTE Mylab twice  Wrist, MCP, PIP, DIP  GS and PD, tenosynovitis 0-3 | Clinical diagnosis | GS:  PIP and DIP of patients with OA revealed score degrees of 1–3 more often compared with the respective joints in the RA cohort. In contrast, more wrist and MCP joints of patients with RA showed inflammatory changes in comparison with those of patients with OA.  PD signal: Patients with RA featured more inflammatory changes in terms of synovitis and tenosynovitis in PD when compared with patients with OA, except for DIP joints in OA. |
| Gok, 2013 [18] | 21 RA / 15 OA | Longstanding RA  Mean (sd) disease duration 6.5 (1.2) years | Cross-sectional study | ESAOTE MyLab 70  Two rheumatologists blinded to clinical data performed US.  The inter-observer agreement for ultrasound examinations was a good level of agreement (k values above 0.821).  Knees  Effusion, GS, PD  (semiquantitative scoring system)  US cumulative activity score: cumulative GS score plus PD score. | Clinical diagnosis | Median (min-max) effusion score in RA 7 (4–9) vs OA 6 (3.5–8.0), p = 0.07.  Median (min-max) synovial hypertrohy score in RA 8 (4–9) vs  OA 5.5 (3.5–8.0), p<0.001.  Median (min-max) PD score in RA 0 (0–4) vs OA 0 (0–4), p>0.05.  Median (min-max) cumulative activity score in RA 15.5 (10.5–20.0) vs OA 11 (7.5–17.0), p<0.001. |
| Hussain, 2018 [19] | 224  RA and OA | RA  Median (IQR) disease duration 42 (117) months | Cross-sectional retrospective study  In the event of any missing information in the outpatient clinic proforma generated in the US clinic at the time of clinical and US examination, the records were completed by consulting the patient electronic hospital records. | GE  Wrist, MCP, PIP  synovial hypertrophy (SH), erosions, PD, effusion, osteophytes, tendon abnormalities  (semiquantitative scoring) | Clinical diagnosis | SH grade 2  Se (95% CI) 0.54 (0.48,0.61)  Sp (95% CI) 0.49 (0.37,0.61)  LR+ (95% CI) 1.07 (0.83,1.39)  LR- (95% CI) 0.92 (0.70,1.21)  PPV (95% CI) 0.01 (0.008,0.1)  NPV (95% CI) 0.99 (0.98,0.99)  SH grade 3  Se (95% CI) 0.68 (0.62,0.74)  Sp (95% CI) 0.44 (0.32,0.56)  LR+ (95% CI) 1.22 (0.97,1.52)  LR- (95% CI) 0.72 (0.52,1.00)  PPV (95% CI) 0.01 (0.01,0.01)  NPV (95% CI) 0.99 (0.99,0.99)  Positive PD  Se (95% CI) 0.92 (0.88,0.95)  Sp (95% CI) 0.47 (0.35,0.59)  LR+ (95% CI) 1.72 (1.38,2.14)  LR- (95% CI) 0.17 (0.10,0.29)  PPV (95% CI) 0.01 (0.01,0.02)  NPV (95% CI) 0.99 (0.99,0.99)  Osteophytes  Se (95% CI) 0.15 (0.10,0.20)  Sp (95% CI) 0.55 (0.43,0.66)  LR+ (95% CI) 0.34 (0.23-0.50)  LR- (95% CI) 1.55 (1.25-1.92)  PPV (95% CI) 0.003 (0.002,0.005)  NPV (95% CI) 0.98 (0.98,0.99)  Erosions  Se (95% CI) 0.58 (0.51,0.64)  Sp (95% CI) 0.70 (0.58,0.80)  LR+ (95% CI) 1.91 (1.32,2.76)  LR- (95% CI) 0.61 (0.49,0.75)  PPV (95% CI) 0.02 (0.01,0.03)  NPV (95% CI) 0.99 (0.99,0.99) |

Summary of findings of articles included in question C2. RA: rheumatoid arthritis; OA: osteoarthritis; GS: grey-scale; PD: power Doppler; SH: synovial hypertrophy; MCP: metacarpophalangeal; PIP: proximal interphalangeal; DIP: distal interphalangeal joints; sd: standard deviation; IQR: interquartile range; CI: confidence interval; Se: sensitivity; Sp: specificity; LR+ positive likelihood ratio; LR- negative likelihood ratio; PPV: positivite predictive value; NPV: negative predictive value; AUC: area under the curve.

Research area D

Supplementary Table S13. In patients with RA, what is the value of level of ultrasound lesion A versus B for outcome?

| **Study** | **N** | **Population** | **Study design** | **Intervention (US technique)** | **Comparator** | **Results** |
| --- | --- | --- | --- | --- | --- | --- |
| Fukae, 2013 [21] | 31 | Longstanding RA starting ADA/TCZ  Mean symptom duration 108.4 months | Prospective cohort study | Hitachi EUP-L34P, EUB-7500, Hitachi  Three experts specialized in musculoskeletal ultrasonography who were blinded to other clinical information  Intraobserver ICC values were 0.997-0.999 for MCP joints and 0.998- 0.999 for PIP joints. The interobserver ICC values were 0.992-0.996 for MCP joints and 0.991-0.999 for PIP joints  MCP and PIP  Quantitative assessment of PD signal, determined by counting the number of vascular flow pixels in the region of interest (ROI) | Joints without synovial vascularity throughout the observational period (0-40 weeks) | ***Radiographic progression – 40 weeks***  *Joints with positive synovial vascularity baseline-week 8:*  ADA group  MCP: no risk of radiographic progression (p=0.497)  PIP: lower risk of progression (p<0.0001)  TCZ group  MCP: no risk of radiographic progression (p=0.744)  PIP: no risk of radiographic progression (p=0.558)  *Joints with intermittent positive synovial vascularity*  ADA group  MCP: higher risk of progression (p=0.0056)  PIP: higher risk of progression (p<0.0001)  TCZ group  MCP: higher risk of progression (p=0.0018)  PIP: higher risk of progression (p=0.0003)  *Joints with persistent positive synovial vascularity*  ADA group  MCP: higher risk of progression (p=0.0035)  PIP: higher risk of progression (p=<0.0001)  TCZ group  MCP: higher risk of progression (p=0.002)  PIP: higher risk of progression (p=0.0112) |
| Gartner, 2013 [22] | 90 | Longstanding RA, one group in CDAI remission and one group with higher levels of disease activity (CDAI >2.8)  Mean disease duration 9.7 years. | Prospective cohort study | GE Logiq E9  An experienced sonographer blinded to clinical data  Intraobserver reliability (assessed by performing the evaluation twice in 10 patients on the same day) revealed a good ICC of 0.978 (0.972 for GS ultrasound and 0.665 for PD ultrasound). The interobserver reliability (using sonographic examinations of identical joints in 10 patients performed by 2 experienced sonographers independently on the same day) revealed good agreement (ICC of 0.856 for all sonographic assessments, 0.837 for GS ultrasound, and 0.762 for PD ultrasound).  Bilateral wrist, MCP, PIP  PD >0 | PD=0 | ***Response***  (Defined as joint swelling)  Patients whose individual joints became clinically swollen at follow-up: higher baseline PD (13 joints [59.1%] no PD, 5 [22.7%] PD grade 1, 4 [18.2%] PD grade >2), as compared to patients whose joints remained unswollen at follow-up (898 joints [80%] no PD, 177 [15.8%] PD grade 1, and 47 [4.2%] showing PD grade >2) (P= 0.0036 by chi-square test) |
| Nordberg, 2018 [20] | 230 (118 in the US strategy group and 112 in the conventional strategy group) | Early RA (<2 years of symptoms duration from the first swollen joint), indication for DMARDs  The mean (±SD) time since patient reported first swollen joint, 6.8 (± 5.2) months in US group, 7.4 (± 5) months in conventional group | Randomized clinical trial  No imputation of missing data was done | Siemens Antares or GE Logiq E9 with linear probes  Trained sonographers  Ultrasound examinations were performed according to a previously published scoring system that has shown good inter- and intraobserver reliability  US of MCPs 1–5, PIP 2-3, radiocarpal, intercarpal, distal radioulnar, elbow, knee, talocrural, and MTPs 1–5  PD score 1-2-3 in non-swollen joints, and PD score 0-1-2-3 in swollen joints | Non-swollen joint with PD score 0 | ***Swollen joint at the next visit in uninjected joints***  Non-swollen joints:  PD score 1 OR (95% CI) 3.6 (2.3,5.5), p<0.001  PD score 2 OR (95% CI) 11.8 (6.9,20.1), p<0.001  PD score 3 OR (95% CI) 12.1 (4.1,35.7), p<0.001  Swollen joints:  PD score 0 OR (95% CI) 5.5 (4.6-6.5), p<0.001  PD score 1 OR (95% CI) 13 (9.4-17.9), p<0.001  PD score 2 OR (95% CI) 31.6 (22.2-45), p<0.001  PD score 3 OR (95% CI) 61 (33.5-111), p<0.001 |
| Raffeiner, 2017 [23] | 121 | Longstanding RA on TNFis, in stable (56 months) DAS28 remission  Mean (± SD) disease duration 14.6 (±8.9) years | Prospective cohort study | Esaote MyLab 70 XVG with a 6-18MHz multifrequency linear transducer  Two sonographers  The interreader agreement for PD location was very high (Cohen’s k = 0.91)  MCPs, PIPs, wrists, MTPs  Total PD score calculated as the sum of PD scores in all joints  A cut-off of a total PD score ≥2 was adopted | PD score <2 | ***Radiographic progression – 1 yr***  (Change in van der Heijde modified total Sharp score>0)  PD score ≥2  RR (95% CI) 3.19 (1.99,5.13) p<0.0001. |
| Ten Cate, 2018 [24] | 174 | Early RA, cs-DMARD naive  Median (IQR) symptom duration 46 (2.8-7.9) months | Prospective cohort study  The analysis with imputed data yielded almost equal results | ESAOTE MyLab60, linear array 6–18 MHz  Seven experienced ultrasonographers blinded to clinical findings  Interobserver reliability: ICC 0.58 (95% CI 0.45–0.72) for GSUS, and 0.48 (0.34–0.64) for PDUS, while it was 0.58 (0.49–0.68) for the two modalities combined  Bilateral MCP 2-5, wrists, MTP 2-5  GS, PD and erosions 0-3 | Different levels of GS and PD | ***Absence of DAS28 remission – 1 yr***  OR (95%) CI  GS>1 0.67(0.27,1.71)  PD>0 0.80 (0.30,2.16)  GS>2 0.56 (0.27,1.20) |

Summary of findings of articles included in question B1. RA: rheumatoid arthritis; GS: grey-scale; PD: power Doppler; MCP: metacarpophalangeal; PIP: proximal interphalangeal; ADA: adalimumab; TCZ: tocilizumab; MTX: methotrexate; LDA: low disease activity; CDAI: clinical disease activity index; OR: odds ratio; RR: risk ratio; sd: standard deviation; IQR: interquartile range; CI: confidence interval

Supplementary Table S14. Studies evaluating the value of level of ultrasound lesion A versus B for outcome. Outcomes of interest are reported in the grey boxes.

| **Study** | **Number of patients** | **Population** | **Intervention (US technique)** | **Comparator** | **Results** |
| --- | --- | --- | --- | --- | --- |
| **Clinical disease activity** | | | | | |
| Nordberg, 2018 [20] | 230 (118 in the US strategy group and 112 in the conventional strategy group) | Early RA  The mean (±SD) time since patient reported first swollen joint, 6.8 (± 5.2) months in US group, 7.4 (± 5) months in conventional group  Follow-up: 24 months  Treatment:  cs-DMARDs and glucocorticoid injections | 32 joints: bilaterally 1-5 MCP, 2-3 PIP, wrist, elbow, knee, talocrural, and 1-5 MTP  PD score 1-2-3 in non-swollen joints, and PD score 0-1-2-3 in swollen joints | Non-swollen joint with PD score 0 | Positive PD score > negative PD score  Higher grade of PD activity increased the risk of clinical disease activity |
| Gärtner, 2013 [22] | 90 | Longstanding RA, one group in CDAI remission and one group in high disease activity (CDAI >2.8)  Mean disease duration 9.7 years  Follow-up: 12 months  Treatment: not specified | 22 joints: bilaterally wrist, 1-5 MCP, 1-5 PIP  PD >0 | PD=0 | Positive PD score > negative PD score  Higher grade of PD activity increased the risk of clinical disease activity |
| **Radiographic progression** | | | | | |
| Fukae, 2013 [21] | 31 | Longstanding RA  Mean symptom duration 108.4 months  Follow-up: 12 months  Treatment: patients treated with DMARDs and with clinical indication to starting Adalimumab or Tocilizumab | 20 joints: bilaterally 1-5 MCP and PIP  Joints with positive synovial vascularity (quantitative assessment of PD signal determined by counting the number of vascular flow pixels in the region of interest) | Joints without synovial vascularity throughout the observational period | Positive synovial vascularity > negative synovial vascularity  Positive synovial vascularity predicts radiographic progression better as no synovial vascularity |
| Raffeiner, 2017 [23] | 121 | Longstanding RA in DAS28 remission  Mean (± SD) disease duration 14.6 (±8.9) years  Follow-up: 12 months  Treatment: cs-DMARDs and/or bDMARDs | 32 joints: bilaterally 1-5 MCP, 1-5 PIP, wrist, 1-5 MTP  Total PD score calculated as the sum of PD scores in all joints. A cut-off of a total PD score ≥2 was adopted | PD score <2 | Higher PD score > lower PD score  Higher PD score predicts radiographic progression better as lower PD score |
| **Absence of clinical remission** | | | | | |
| Ten Cate, 2018 [24] | 174 | Early RA, cs-DMARD naïve  Median (IQR) symptom duration 46 (2.8-7.9) months  Follow-up: 12 months  Treatment: cs-DMARDs and/or bDMARDs | 18 joints: bilaterally MCP 2-5, wrist, MTP 2-5  GS >1  PD>0 | GS ≤ 1  PD =0 | Higher GS or PD scores = lower GS or PD scores  Higher GS or PD scores predict the absence of DAS28 remission in the same way as lower GS or PD scores |

Abbreviations: US: ultrasound; RA: rheumatoid arthritis; MCP: metacarpophalangeal; PIP: proximal interphalangeal; MTP: metatarsophalangeal; PD: power Doppler; cs-DMARDS: conventional synthetic disease-modifying antirheumatic drugs; bDMARDs: biologic disease modifying antirheumatic drugs; CDAI: clinical disease activity index; MTX: methotrexate; TNFi: tumor necrosis factor inhibitor; LDA: low disease activity; DAS28: disease activity score on 28 joints; IQR: interquartile range.

**References**

1. Naredo E, Rodríguez M, Campos C, Rodríguez-Heredia JM, Medina JA, Giner E, et al. Validity, reproducibility, and responsiveness of a twelve-joint simplified power doppler ultrasonographic assessment of joint inflammation in rheumatoid arthritis. Arthritis Rheum. 2008;59:515–22.

2. Naredo E, Valor L, De la Torre I, Martínez-Barrio J, Hinojosa M, Aramburu F, et al. Ultrasound joint inflammation in rheumatoid arthritis in clinical remission: how many and which joints should be assessed? Arthritis Care Res. 2013;65:512–7.

3. Picchianti Diamanti A, Navarini L, Messina F, Markovic M, Arcarese L, Basta F, et al. Ultrasound detection of subclinical synovitis in rheumatoid arthritis patients in clinical remission: a new reduced-joint assessment in 3 target joints. Clin. Exp. Rheumatol. 2018;36:984–9.

4. Dougados M, Jousse-Joulin S, Mistretta F, d’Agostino M-A, Backhaus M, Bentin J, et al. Evaluation of several ultrasonography scoring systems for synovitis and comparison to clinical examination: results from a prospective multicentre study of rheumatoid arthritis. Ann. Rheum. Dis. 2010;69:828–33.

5. El-Gohary RM, Ahmed Mahmoud AA-M, Khalil A, El-Gendy H, Gado KH. Validity of 7-Joint Versus Simplified 12-Joint Ultrasonography Scoring Systems in Assessment of Rheumatoid Arthritis Activity. J. Clin. Rheumatol. Pract. Rep. Rheum. Musculoskelet. Dis. 2019;25:264–71.

6. Hammer HB, Kvien TK. Comparisons of 7- to 78-joint ultrasonography scores: all different joint combinations show equal response to adalimumab treatment in patients with rheumatoid arthritis. Arthritis Res. Ther. 2011;13:R78.

7. Kawashiri S, Kawakami A, Iwamoto N, Fujikawa K, Satoh K, Tamai M, et al. The power Doppler ultrasonography score from 24 synovial sites or 6 simplified synovial sites, including the metacarpophalangeal joints, reflects the clinical disease activity and level of serum biomarkers in patients with rheumatoid arthritis. Rheumatol. Oxf. Engl. 2011;50:962–5.

8. Naredo E., Gamero F., Bonilla G., Uson J., Carmona L., Laffon A. Ultrasonographic assessment of inflammatory activity in rhematoid arthritis: Comparison of extended versus reduced joint evaluation. Clin. Exp. Rheumatol. 2005;23:881–4.

9. Fukae J, Shimizu M, Kon Y, Tanimura K, Matsuhashi M, Kamishima T, et al. Screening for rheumatoid arthritis with finger joint power Doppler ultrasonography: quantification of conventional power Doppler ultrasonographic scoring. Mod. Rheumatol. 2009;19:502–6.

10. Tan YK, Allen JC, Lye WK, Chew L-C, Thumboo J. Greater rheumatoid arthritis joint improvement with more subjects achieving response across improvement categories using novel versus existing ultrasound methods. Rheumatol. Int. 2018;38:795–9.

11. Kawahara R., Nakabo S., Shimizu M., Yamamoto H., Sasai T., Nishida Y., et al. Feasibility of patient-oriented ultrasound joint selection: Cross-sectional observational study on rheumatoid arthritis. Mod. Rheumatol. [Internet] 2019;Available from: http://www.embase.com/search/results?subaction=viewrecord&from=export&id=L2003524516

12. Sivakumaran P, Hussain S, Attipoe L, Ciurtin C. Diagnostic accuracy of simplified ultrasound hand examination protocols for detection of inflammation and disease burden in patients with rheumatoid arthritis. Acta Radiol. Stockh. Swed. 1987 2019;60:92–9.

13. Ellegaard K., Terslev L., Christensen R., Szkudlarek M., Schmidt W.A., Jensen P.S., et al. Comparison of discrimination and prognostic value of two US Doppler scoring systems in rheumatoid arthritis patients: A prospective cohort study. Clin. Exp. Rheumatol. 2014;32:495–500.

14. Fukae J, Kon Y, Henmi M, Sakamoto F, Narita A, Shimizu M, et al. Change of synovial vascularity in a single finger joint assessed by power doppler sonography correlated with radiographic change in rheumatoid arthritis: comparative study of a novel quantitative score with a semiquantitative score. Arthritis Care Res. 2010;62:657–63.

15. Janta I, Valor L, De la Torre I, Martínez-Estupiñán L, Nieto JC, Ovalles-Bonilla JG, et al. Ultrasound-detected activity in rheumatoid arthritis on methotrexate therapy: Which joints and tendons should be assessed to predict unstable remission? Rheumatol. Int. 2016;36:387–96.

16. Ehrenstein B, Pongratz G, Fleck M, Hartung W. The ability of rheumatologists blinded to prior workup to diagnose rheumatoid arthritis only by clinical assessment: a cross-sectional study. Rheumatol. Oxf. Engl. 2018;57:1592–601.

17. Glimm A-M, Werner SG, Burmester GR, Backhaus M, Ohrndorf S. Analysis of distribution and severity of inflammation in patients with osteoarthitis compared to rheumatoid arthritis by ICG-enhanced fluorescence optical imaging and musculoskeletal ultrasound: a pilot study. Ann. Rheum. Dis. 2016;75:566–70.

18. Gok M, Erdem H, Gogus F, Yilmaz S, Karadag O, Simsek I, et al. Relationship of ultrasonographic findings with synovial angiogenesis modulators in different forms of knee arthritides. Rheumatol. Int. 2013;33:879–85.

19. Hussain S, Sivakumaran P, Gill A, Dhas D, Ciurtin C. Ultrasonography-detected subclinical inflammation in patients with hand osteoarthritis and established rheumatoid arthritis: a comparison between two different pathologies using the same ultrasound examination protocol. Musculoskeletal Care 2018;16:26–31.

20. Nordberg LB, Lillegraven S, Aga A-B, Sexton J, Lie E, Hammer HB, et al. The Impact of Ultrasound on the Use and Efficacy of Intraarticular Glucocorticoid Injections in Early Rheumatoid Arthritis: Secondary Analyses From a Randomized Trial Examining the Benefit of Ultrasound in a Clinical Tight Control Regimen. Arthritis Rheumatol. Hoboken NJ 2018;70:1192–9.

21. Fukae J, Isobe M, Kitano A, Henmi M, Sakamoto F, Narita A, et al. Positive synovial vascularity in patients with low disease activity indicates smouldering inflammation leading to joint damage in rheumatoid arthritis: time-integrated joint inflammation estimated by synovial vascularity in each finger joint. Rheumatol. Oxf. Engl. 2013;52:523–8.

22. Gärtner M, Mandl P, Radner H, Supp G, Machold KP, Aletaha D, et al. Sonographic joint assessment in rheumatoid arthritis: associations with clinical joint assessment during a state of remission. Arthritis Rheum. 2013;65:2005–14.

23. Raffeiner B, Grisan E, Botsios C, Stramare R, Rizzo G, Bernardi L, et al. Grade and location of power Doppler are predictive of damage progression in rheumatoid arthritis patients in clinical remission by anti-tumour necrosis factor α. Rheumatol. Oxf. Engl. 2017;56:1320–5.

24. Ten Cate DF, Jacobs JWG, Swen W a. A, Hazes JMW, de Jager MH, Basoski NM, et al. Can baseline ultrasound results help to predict failure to achieve DAS28 remission after 1 year of tight control treatment in early RA patients? Arthritis Res. Ther. 2018;20:15.

25. Brown AK, Conaghan PG, Karim Z, Quinn MA, Ikeda K, Peterfy CG, et al. An explanation for the apparent dissociation between clinical remission and continued structural deterioration in rheumatoid arthritis. Arthritis Rheum. 2008;58:2958–67.

26. Chen Y-C, Su F-M, Hsu S-W, Chen J-F, Cheng T-T, Lai H-M, et al. Predictor of Hand Radiological Progression in Patients With Rheumatoid Arthritis Receiving TNF Antagonist Therapy by Change in Grayscale Synovitis-A Preliminary Study. J. Clin. Rheumatol. Pract. Rep. Rheum. Musculoskelet. Dis. 2017;23:73–6.

27. de Miguel E, Pecondón-Español A, Castaño-Sánchez M, Corrales A, Gutierrez-Polo R, Rodriguez-Gomez M, et al. A reduced 12-joint ultrasound examination predicts lack of X-ray progression better than clinical remission criteria in patients with rheumatoid arthritis. Rheumatol. Int. 2017;37:1347–56.

28. Di Carlo M., Salaffi F., Gremese E., Iannone F., Lapadula G., Ferraccioli G. Body mass index as a driver of selection of biologic therapy in rheumatoid arthritis. Results from the US-CLARA study. Eur. J. Intern. Med. 2019;66:57–61.

29. Dougados M, Devauchelle-Pensec V, Ferlet JF, Jousse-Joulin S, D’Agostino M-A, Backhaus M, et al. The ability of synovitis to predict structural damage in rheumatoid arthritis: a comparative study between clinical examination and ultrasound. Ann. Rheum. Dis. 2013;72:665–71.

30. Ellegaard K, Christensen R, Torp-Pedersen S, Terslev L, Holm CC, Kønig MJ, et al. Ultrasound Doppler measurements predict success of treatment with anti-TNF-&alpha; drug in patients with rheumatoid arthritis: a prospective cohort study. Rheumatol. Oxf. Engl. 2011;50:506–12.

31. Filippou G, Sakellariou G, Scirè CA, Carrara G, Rumi F, Bellis E, et al. The predictive role of ultrasound-detected tenosynovitis and joint synovitis for flare in patients with rheumatoid arthritis in stable remission. Results of an Italian multicentre study of the Italian Society for Rheumatology Group for Ultrasound: the STARTER study. Ann. Rheum. Dis. 2018;77:1283–9.

32. Fisher BA, Donatien P, Filer A, Winlove CP, McInnes IB, Buckley CD, et al. Decrease in articular hypoxia and synovial blood flow at early time points following infliximab and etanercept treatment in rheumatoid arthritis. Clin. Exp. Rheumatol. 2016;34:1072–6.

33. Foltz V, Gandjbakhch F, Etchepare F, Rosenberg C, Tanguy ML, Rozenberg S, et al. Power Doppler ultrasound, but not low-field magnetic resonance imaging, predicts relapse and radiographic disease progression in rheumatoid arthritis patients with low levels of disease activity. Arthritis Rheum. 2012;64:67–76.

34. Geng Y, Han J, Deng X, Zhang Z. Deep clinical remission: an optimised target in the management of rheumatoid arthritis? Experience from an ultrasonography study. Clin. Exp. Rheumatol. 2016;34:581–6.

35. Harman H., Tekeoglu I. Clinical and ultrasonographic findings in patients with early rheumatoid arthritis: An 18-month follow-up cohort study. Med. J. Bakirkoy 2018;14:155–64.

36. Harman H, Tekeoğlu İ, Takçı S, Kamanlı A, Nas K, Harman S. Improvement of large-joint ultrasonographic synovitis is delayed in patients with newly diagnosed rheumatoid arthritis: results of a 12-month clinical and ultrasonographic follow-up study of a local cohort. Clin. Rheumatol. 2015;34:1367–74.

37. Horton SC, Tan AL, Freeston JE, Wakefield RJ, Buch MH, Emery P. Discordance between the predictors of clinical and imaging remission in patients with early rheumatoid arthritis in clinical practice: implications for the use of ultrasound within a treatment-to-target strategy. Rheumatol. Oxf. Engl. 2016;55:1177–87.

38. Ikeda K, Nakagomi D, Sanayama Y, Yamagata M, Okubo A, Iwamoto T, et al. Correlation of radiographic progression with the cumulative activity of synovitis estimated by power Doppler ultrasound in rheumatoid arthritis: difference between patients treated with methotrexate and those treated with biological agents. J. Rheumatol. 2013;40:1967–76.

39. Inanc N., Ozen G., Direskeneli H. Predictive value of ultrasonographic assessment of disease activity in response to tumour necrosis factor-a inhibitor treatment in rheumatoid arthritis: A prospective cohort study. Clin. Exp. Rheumatol. 2016;34:156–156.

40. Iwamoto T, Ikeda K, Hosokawa J, Yamagata M, Tanaka S, Norimoto A, et al. Prediction of relapse after discontinuation of biologic agents by ultrasonographic assessment in patients with rheumatoid arthritis in clinical remission: high predictive values of total gray-scale and power Doppler scores that represent residual synovial inflammation before discontinuation. Arthritis Care Res. 2014;66:1576–81.

41. Kawashiri S-Y, Fujikawa K, Nishino A, Okada A, Aramaki T, Shimizu T, et al. Ultrasound-detected bone erosion is a relapse risk factor after discontinuation of biologic disease-modifying antirheumatic drugs in patients with rheumatoid arthritis whose ultrasound power Doppler synovitis activity and clinical disease activity are well controlled. Arthritis Res. Ther. 2017;19:108.

42. Matsuo H., Imamura A., Shimizu M., Inagaki M., Tsuji Y., Nakabo S., et al. Prediction of recurrence and remission using superb microvascular imaging in rheumatoid arthritis. J. Med. Ultrason. 2020;47:131–8.

43. Naredo E, Collado P, Cruz A, Palop MJ, Cabero F, Richi P, et al. Longitudinal power Doppler ultrasonographic assessment of joint inflammatory activity in early rheumatoid arthritis: predictive value in disease activity and radiologic progression. Arthritis Rheum. 2007;57:116–24.

44. Naredo E, Valor L, De la Torre I, Montoro M, Bello N, Martínez-Barrio J, et al. Predictive value of Doppler ultrasound-detected synovitis in relation to failed tapering of biologic therapy in patients with rheumatoid arthritis. Rheumatol. Oxf. Engl. 2015;54:1408–14.

45. Saleem B., Brown A.K., Quinn M., Karim Z., Hensor E.M.A., Conaghan P., et al. Can flare be predicted in DMARD treated RA patients in remission, and is it important? A cohort study. Ann. Rheum. Dis. 2012;71:1316–21.

46. Sapundzhieva T, Karalilova R, Batalov A. Musculoskeletal ultrasound as a biomarker of remission - results from a one-year prospective study in patients with rheumatoid arthritis. Med. Ultrason. 2018;20:453–60.

47. Scirè CA, Montecucco C, Codullo V, Epis O, Todoerti M, Caporali R. Ultrasonographic evaluation of joint involvement in early rheumatoid arthritis in clinical remission: power Doppler signal predicts short-term relapse. Rheumatol. Oxf. Engl. 2009;48:1092–7.

48. Lamers-Karnebeek FB, Luime JJ, Ten Cate DF, Teerenstra S, Swen NWAA, Gerards AH, et al. Limited value for ultrasonography in predicting flare in rheumatoid arthritis patients with low disease activity stopping TNF inhibitors. Rheumatol. Oxf. Engl. 2017;56:1560–5.

49. Paulshus Sundlisæter N, Aga A-B, Olsen IC, Hammer HB, Uhlig T, van der Heijde D, et al. Clinical and ultrasound remission after 6 months of treat-to-target therapy in early rheumatoid arthritis: associations to future good radiographic and physical outcomes. Ann. Rheum. Dis. 2018;77:1421–5.

50. Paulshus Sundlisæter N, Olsen IC, Aga A-B, Hammer HB, Uhlig T, van der Heijde D, et al. Predictors of sustained remission in patients with early rheumatoid arthritis treated according to an aggressive treat-to-target protocol. Rheumatol. Oxf. Engl. 2018;57:2022–31.

51. Abdelzaher MG, Tharwat S, AbdElkhalek A, Abdelsalam A. Ultrasound versus magnetic resonance imaging in the evaluation of shoulder joint pathologies in a cohort of rheumatoid arthritis patients. Int. J. Rheum. Dis. 2019;22:2158–64.

52. Damjanov N, Radunovic G, Prodanovic S, Vukovic V, Milic V, Simic Pasalic K, et al. Construct validity and reliability of ultrasound disease activity score in assessing joint inflammation in RA: comparison with DAS-28. Rheumatol. Oxf. Engl. 2012;51:120–8.

53. EL-Melegy D.N., El-Khouly R.M., Mwafi M.E.E.-D., Zyton H.A.E.-H. Magnetic resonance imaging versus musculoskeletal ultrasound in the evaluation of temporomandibular joint in rheumatoid arthritis patients. Egypt. Rheumatol. 2017;39:207–11.

54. Ogishima H, Tsuboi H, Umeda N, Horikoshi M, Kondo Y, Sugihara M, et al. Analysis of subclinical synovitis detected by ultrasonography and low-field magnetic resonance imaging in patients with rheumatoid arthritis. Mod. Rheumatol. 2014;24:60–8.

55. Taniguchi D, Tokunaga D, Oda R, Fujiwara H, Ikeda T, Ikoma K, et al. Maximum intensity projection with magnetic resonance imaging for evaluating synovitis of the hand in rheumatoid arthritis: comparison with clinical and ultrasound findings. Clin. Rheumatol. 2014;33:911–7.

56. Zou H., Beattie K.A., Allen M., Ioannidis G., Larché M.J. Ultrasonography supplements clinical exam to improve early rheumatoid arthritis disease activity monitoring in metatarsophalangeal joints. Clin. Rheumatol. 2020;39:1483–91.

57. Funck-Brentano T, Gandjbakhch F, Etchepare F, Jousse-Joulin S, Miquel A, Cyteval C, et al. Prediction of radiographic damage in early arthritis by sonographic erosions and power Doppler signal: a longitudinal observational study. Arthritis Care Res. 2013;65:896–902.

58. Nozaki Y., Ri J., Sakai K., Shiga T., Inoue A., Nagare Y., et al. Prediction of therapeutic responses with ultrasonography in RA patients treated with an anti-TNF drug: a retrospective cohort study. Immunol. Med. 2018;41:129–35.

59. Ramírez J, Narváez JA, Ruiz-Esquide V, Hernández-Gañán J, Cuervo A, Inciarte-Mundo J, et al. Clinical and sonographic biomarkers of structural damage progression in RA patients in clinical remission: A prospective study with 12 months follow-up. Semin. Arthritis Rheum. 2017;47:303–9.

60. Saleem B., Keen H., Goeb V., Parmar R., Nizam S., Hensor E.M.A., et al. Patients with RA in remission on TNF blockers: When and in whom can TNF blocker therapy be stopped? Ann. Rheum. Dis. 2010;69:1636–42.
